# Chalcone Derivatives: Antioxidant Activity, Photoprotective Properties, and Stability under UV Irradiation

**DOI:** 10.1021/acsomega.5c08934

**Published:** 2025-12-15

**Authors:** Raphaela P. Guaringue, Vinicius M. Schaffka, Larissa Kozan, André L. Kerek, Eduardo Protachevicz, André V. Bassani, Christiana A. Pessoa, Patrícia M. Campos, Guilherme A. Camargo, Barbara C. Fiorin

**Affiliations:** † Chemistry Department, State University of Ponta Grossa, Av. Carlos Cavalcanti, 4748, Ponta Grossa 84030-900, Brazil; ‡ Department of Pharmaceutical Sciences, State University of Ponta Grossa, Av. Carlos Cavalcanti, 4748, Ponta Grossa 84030-900, Brazil; § Department of Pharmacy, Federal University of Paraná, Av. Prefeito Lothário Meissner, 632, Curitiba 80210-170, Brazil

## Abstract

The development of multifunctional photoprotective agents capable of combining ultraviolet (UV) radiation absorption, photostability, and antioxidant activity represents a major challenge in cosmetic science. In this study, a series of 14 chalcone derivatives was synthesized and evaluated for their antioxidant and photoprotective potential. The chalcones, synthesized via the Claisen–Schmidt condensation, were evaluated for their antioxidant activity through DPPH, FRAP, and cyclic voltammetry assays, revealing that compounds 6, 7, and 12 exhibited the highest free radical scavenging capacity, reducing power, and inhibition of lipid peroxidation, with electrochemical data confirming facile oxidation via proton-coupled electron transfer. Photoprotective assessment by the Mansur method demonstrated that these chalcones showed moderate sun protection factor values, with absorption in both UVA and UVB regions and critical wavelengths (λ_c_) compatible with broad-spectrum filters. Photostability assays under prolonged UVA irradiation highlighted Chalcone 6 (Chal 6) as exceptionally stable, maintaining its initial absorbance and complete structural integrity even after 72 h of continuous exposure, as monitored by ^1^H NMR. Other chalcones underwent partial *trans–cis* isomerization accompanied by reduced absorbance. The remarkable stability of Chal 6, combined with its antioxidant profile, suggests its potential as a promising candidate for multifunctional sunscreen formulations capable of overcoming the current limitations of unstable UVA filters.

## Graphical abstract

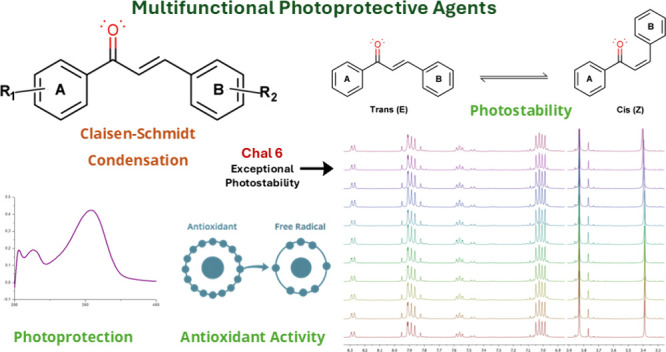

## Introduction

Excessive exposure to solar ultraviolet (UV) radiation is one of the main environmental risk factors for skin damage, photoaging, and the development of skin cancers. UVB radiation (280–320 nm) is primarily responsible for inducing erythema and direct DNA damage, whereas UVA radiation (320–400 nm) penetrates more deeply into the dermis, contributing to oxidative stress, collagen degradation, and long-term skin aging.^1,2^ Although sunscreens are widely used as the first line of defense against UV-induced damage, there remains a particular challenge in effectively blocking UVA radiation. Currently, only a few UVA filters are approved for cosmetic use, and many of them exhibit low photostability, which results in a loss of efficacy during sun exposure.^1,3^

Among the currently used UVA filters, avobenzone (butyl methoxydibenzoylmethane) is one of the most effective; however, its application is strongly limited by its well-known photoinstability. Under UV irradiation, avobenzone undergoes photochemical reactions that generate free radicals and reactive oxygen species (ROS), along with unstable degradation products, ultimately reducing its photoprotective efficacy.^4^ Other organic filters raise environmental and health concerns, such as oxybenzone, which has been linked to coral bleaching and endocrine disruption.^5^ Inorganic filters, such as TiO_2_ and ZnO, are photostable but often present aesthetic drawbacks due to their whitening effect. These limitations highlight the need for the development of novel UVA filters that are both stable and effective.^6^

Antioxidant activity in sunscreens is crucial, as even partial UV radiation penetration can reach deeper skin layers, generating ROS that damage lipids, proteins, and DNA. The inclusion of antioxidant compounds in the formulation provides a secondary line of defense by neutralizing free radicals and preventing inflammatory and degenerative processes associated with photoaging.^7^

Thus, there is growing interest in multifunctional bioactive molecules capable of absorbing UVA and UVB radiation while simultaneously neutralizing ROS. Phenolic compounds, especially flavonoids, have attracted attention due to their strong absorption in the UV spectrum and high antioxidant capacity. Among them, chalcones, precursors of flavonoids characterized by an α,β-unsaturated carbonyl system linking two aromatic rings (C6–C3–C6) (Figure 1), show a distinctive potential for use as sunscreen agents, combining UVA/UVB absorption, high photostability, and potent antioxidant activity.^8,9^

**Figure 1:**
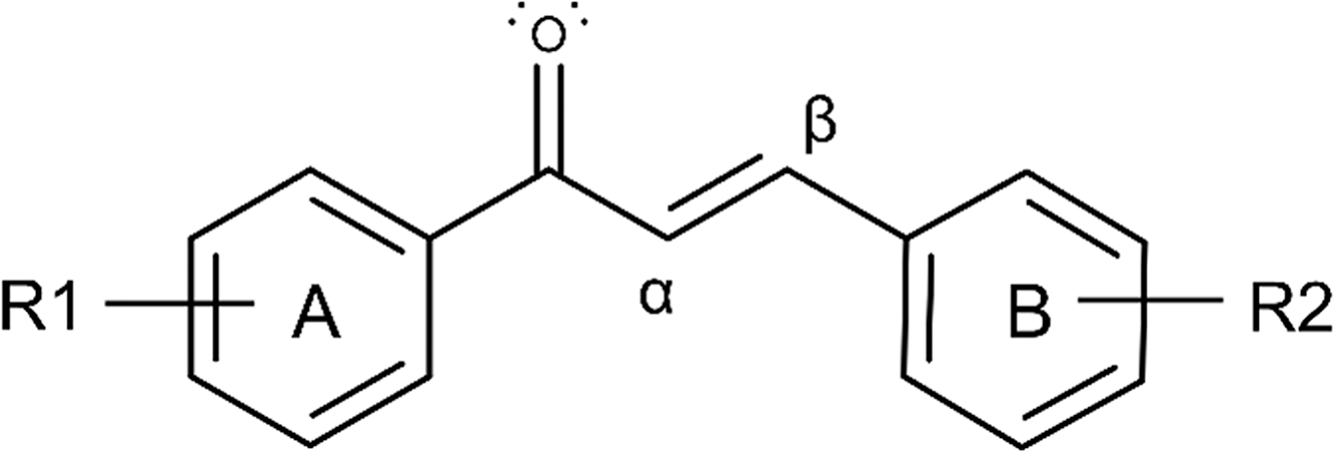
General chemical structure of chalcones (C6–C3–C6 system).

Several natural chalcones, such as licochalcone A from *Glycyrrhiza inflata*, have already been incorporated into commercial sunscreens due to their ability to reduce UV-induced oxidative stress and inflammation.^10^ Structural modifications, such as methoxylation or the introduction of heterocycles, can further enhance photostability and lipophilic affinity, favoring their incorporation into cosmetic formulations.^11,12^ Despite these promising attributes, there are still few investigations on structure–activity relationships (SAR) of chalcones specifically focused on UVA protection combined with antioxidant activity.

In this study, we report the synthesis, structural characterization, antioxidant evaluation, and in vitro photoprotective assessment of a series of chalcone derivatives, aiming to identify compounds with a multifunctional photoprotective profile capable of meeting the demand for stable and effective UVA filters with significant antioxidant activity in the market.

## Results and Discussion

### Synthesis and Characterization

The synthetic methodology employed proved to be efficient for obtaining the desired derivatives. The purity and structural identity of all compounds were confirmed by melting point determination and by ^1^H and ^13^C NMR spectroscopy.

All compounds (1–14) (Figure 2) were obtained as pure solids and fully characterized. The *trans* configuration of the α,β-unsaturated double bond was confirmed for all derivatives by the characteristic coupling constants (*J* = 15.4–16.1 Hz) between the vinylic protons in the ^1^H NMR spectra.

**Figure 2:**
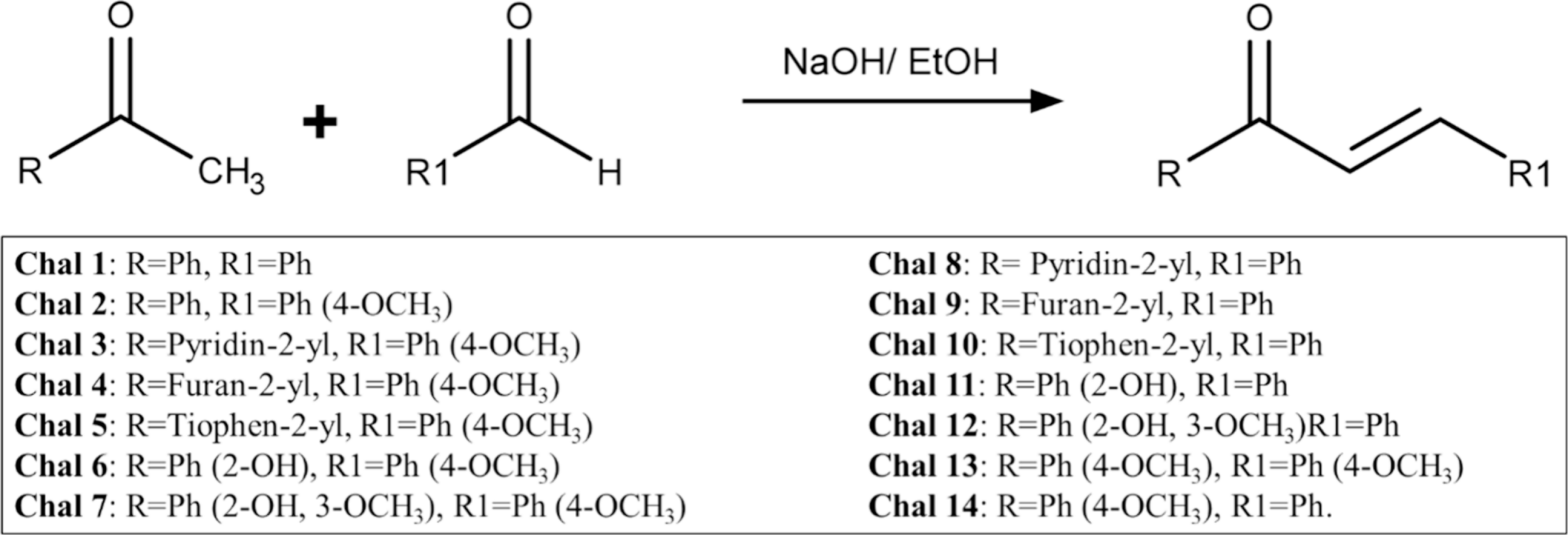
Chalcone synthesis scheme (Chal 1–14) via the Claisen–Schmidt reaction.

These results confirm the efficient synthesis and full characterization of all chalcone derivatives, with the complete NMR spectra provided in the Supporting Information (Figures S1–S28).

### Antioxidant Activity

The antioxidant potential of the synthesized chalcones was evaluated by DPPH radical scavenging, ferric-reducing antioxidant power (FRAP), and electrochemical assay, providing complementary information on radical scavenging, reducing ability, and redox behavior.

#### DPPH Radical Scavenging Assay

The DPPH assay measures the reduction of the violet-colored DPPH radical (λ_max_ = 517 nm) by hydrogen or electron donation, indicated by a decrease in the absorbance. Among the chalcones, Chal 6, 7, and 12 showed the highest activity, with inhibition values of 77.5, 79.3, and 72.3% and IC_5__0_ values of 3.78, 10.06, and 23.73 mmol·L^–1^, respectively (Table 1). This high activity correlates with the presence of hydroxyl and methoxy groups, which facilitate hydrogen donation and stabilize the radical species.^3^

**Table 1:** Antioxidant Activity of Chalcones as Measured by the DPPH Radical Inhibition Assay, Expressed as % Inhibition and IC_5__0_ (mmol·L^–1^)­^a^

Compound	% Inhibition (50 mmol·L^–1^)	IC_50_ (mmol·L^–1^)
Chal 1	22.81 ± 4.35^a^	>50
Chal 2	15.14 ± 3.61^a^	>50
Chal 3	59.80 ± 1.45^b^	38.48
Chal 4	48.56 ± 4.16^c^	>50
Chal 5	36.24 ± 0.88^c^	>50
Chal 6	77.51 ± 0.75^b^	3.78
Chal 7	79.30 ± 4.89^b^	10.06
Chal 8	30.76 ± 3.27^a^	>50
Chal 9	36.18 ± 6.82^c^	>50
Chal 10	9.14 ± 5.86^a^	>50
Chal 11	29.32 ± 1.75^a,c^	>50
Chal 12	72.29 ± 2.29^b^	23.73
Chal 13	46.17 ± 2.65^c^	>50
Chal 14	0.00^e^	>50
Ascorbic acid	100 ± 0.46	1.78

a Mean ± SD (*n* = 3). Different letters (a–e) indicate significant differences by Tukey’s test (*p* < 0.05).

#### FRAP Assay

The FRAP assay measures the ability to reduce Fe^3^^+^ to Fe^2^^+^. Consistently, Chal 6, 7, and 12 exhibited the highest reducing power with values of 30.48, 33.76, and 19.94 mmol Fe^2^^+^·mmol^–1^, respectively (Table 2). The presence of hydroxyl groups in the aromatic rings provides labile electrons for reduction, while methoxy substituents enhance resonance stabilization, improving the redox process. Chalcones lacking hydroxyl groups, such as Chal 2, displayed minimal activity. These results corroborate previous findings that hydroxylated chalcones possess superior reducing power compared to methoxylated chalcones.^12^

**Table 2:** Chalcone Reducing Capacity Determined by the FRAP Assay, Expressed in mmol Fe^2^^+^ Equivalents per mmol of Sample^a^

**Compound**	**mmol eq Fe** **^2+^** **·mmol sample** ^**–1**^
Chal 1	0.40 ± 0.03^a^
Chal 2	0.00^b^
Chal 3	8.65 ± 0.15^c^
Chal 4	0.56 ± 0.21^a^
Chal 5	0.00^b^
Chal 6	30.48 ± 0.16^d^
Chal 7	33.76 ± 0.38^e^
Chal 8	2.08 ± 0.24^f^
Chal 9	0.52 ± 0.13^a^
Chal 10	4.05 ± 0.15^g^
Chal 11	0.94 ± 0.05^a,g^
Chal 12	19.94 ± 0.65^h^
Chal 13	6.23 ± 0.69^i^
Chal 14	1.58 ± 0.35^f^

a Mean ± SD (*n* = 3). Different letters (a–i) indicate significant differences by Tukey’s test (*p* < 0.05).

#### Electrochemical Assay

Electrochemical methods provide valuable insights into electron transfer reactions without prior sample preparation, offering rapid and highly sensitive responses.^13^ In antioxidant evaluation, the oxidation potential (*E*_p__a_) is a key parameter: lower *E*_p__a_ values reflect an enhanced ability to donate electrons, correlating with higher antioxidant activity depending on the radical species involved.^14^

Initial screening of Chal 1 (Figure S29) revealed no oxidation process within the studied potential window and pH range, suggesting that the unsubstituted chalcone scaffold lacks significant electroactivity under these conditions.

In contrast, well-defined anodic responses were observed for Chal 6, 7, 11, and 12 in all tested pH values (Figure 3). These active compounds share a common feature: hydroxyl substituents, which are known for their antioxidant properties and their ability to stabilize radical intermediates.^15,16^

**Figure 3:**
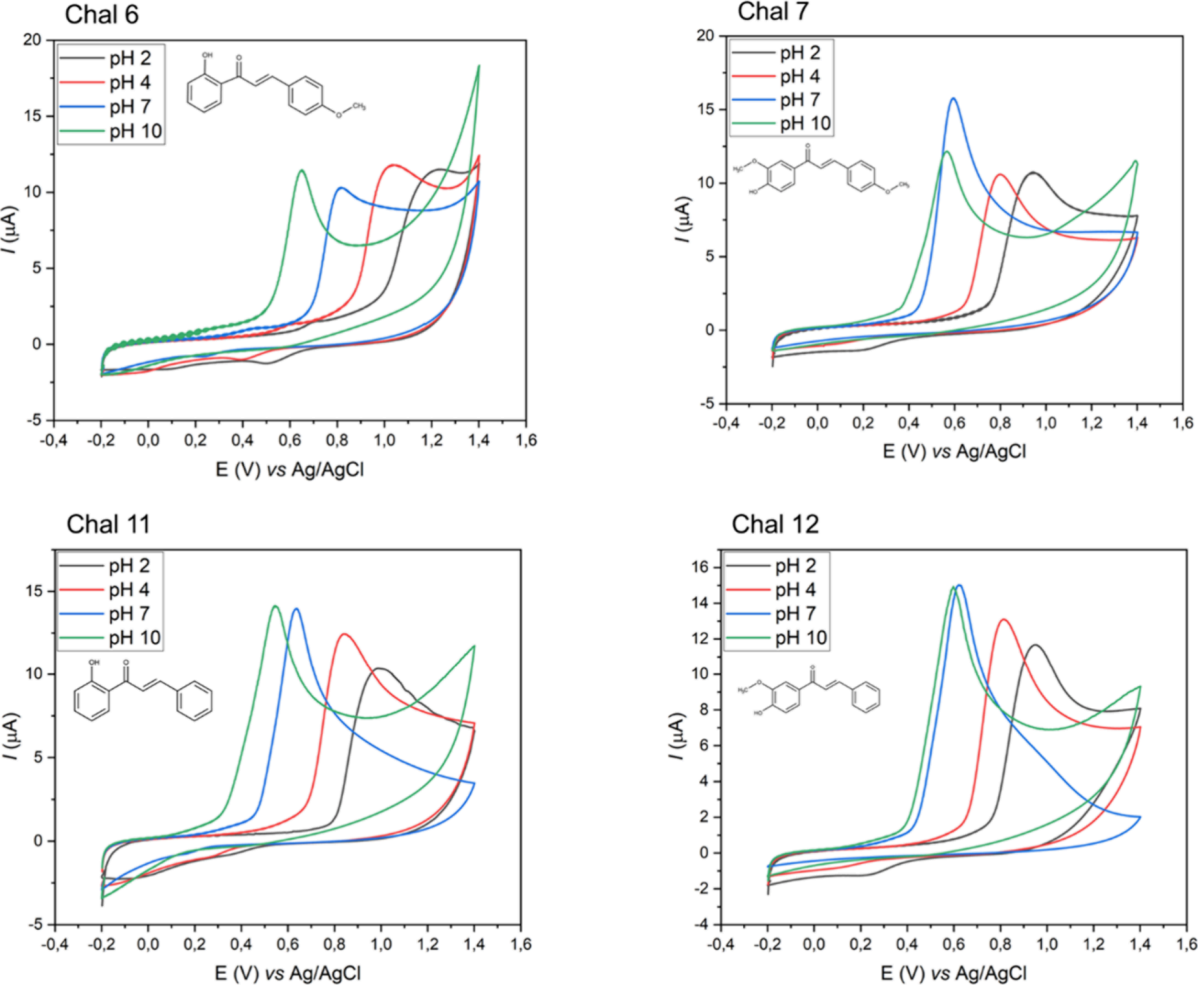
Cyclic voltammograms of compounds Chal 6, 7, 11, and 12 in BR buffer 0.04 M pH 2–10 and ethanol 1:1 (v/v), in the potential range of −0.2 to 1.4 V, with a scan rate of 0.1 V s^–1^.

The oxidation of phenolic compounds occurs via an electron transfer mechanism, generating phenoxyl radicals. These intermediates can undergo dimerization or polycondensation reactions, leading to stabilization of the oxidized species and reduced reactivity. Resonance delocalization of the phenoxyl radical within the aromatic ring facilitates oxidation, resulting in lower *E*_p__a_ values.^17,18^

As shown in Table 3, *E*_p__a_ values decreased progressively with increasing pH, indicating that alkaline media enhance antioxidant efficiency. At higher pH, phenolic groups are deprotonated to form phenolates, which are stronger nucleophiles and more effective electron donors than neutral phenols.^19^ A linear relationship between pH and *E*_p__a_ was confirmed for all active chalcones (Figure S30), consistent with a proton-coupled electron transfer (PCET) mechanism, commonly observed in phenolic antioxidant oxidation.^20^

**Table 3:** *E*_pa_ Values Obtained for Different pHs for Chal 6, 7, 11, and 12

	*E*_pa_ vs Ag/AgCl			
pH	Chal 6	Chal 7	Chal 11	Chal 12
2	1.18	0.94	0.99	0.96
4	1.03	0.80	0.84	0.81
7	0.82	0.61	0.63	0.63
10	0.65	0.55	0.54	0.59

The experimental slopes from *E* vs pH plots were close to the theoretical value of 0.059 V·pH^–1^ predicted by the Nernst equation (eqs 1 and 2), indicating equimolar proton and electron participation during the redox process:

$$E\;=\;\frac{-RT}{nF}\mathrm{pH}$$ 1

At 25 °C, this simplifies to

$$E\;=\;\frac{-0.059}{n}\mathrm{pH}$$ 2

As summarized in Table 4, high correlation coefficients (*R* ^2^ > 0.94) confirm the robustness of the PCET process. Chalcone 6 showed both the lowest oxidation potentials and a slope closest to the theoretical value, reflecting efficient and stable redox activity in the tested pH range.

**Table 4:** Linear Equations Obtained from the Relation *E* vs pH

**Compound**	**Equation**	***R* ^2^**
Chal 6	–0.066 pH + 1.30	0.996
Chal 7	–0.050 pH + 1.01	0.941
Chal 11	–0.057 pH + 1.08	0.967
Chal 12	–0.048 pH + 1.02	0.949

Overall, these findings demonstrate that hydroxyl-substituted chalcones, particularly Chal 6, possess a favorable redox profile compatible with strong antioxidant activity. The electrochemical behavior correlates with their chemical antioxidant performance (DPPH and FRAP), reinforcing their potential application as radical scavengers in cosmetic and pharmaceutical formulations, especially for mitigating UV-induced oxidative stress.

The consistent performances of Chal 6, 7, and 12 obtained in all assays highlight the synergistic effect of hydroxyl and methoxy substituents. Hydroxyl groups provide hydrogen atoms for radical scavenging and redox reactions, while methoxy groups stabilize the aromatic system through resonance, enhancing electron density and facilitating radical neutralization. Chalcones without hydroxyl groups or with sterically hindered substituents displayed lower activity, demonstrating the critical influence of functional group identity and position on antioxidant efficacy.^17,21^

### Photoprotective Activity

The search for organic sunscreens with high efficiency in the UVA region represents a significant challenge in photoprotection given the need to combine absorption at longer wavelengths with adequate photochemical stability. Our results (Figure 4) demonstrate that the presence of a methoxy group (−OCH_3_) at the *para* position of ring B is an effective molecular strategy to optimize the photoprotective properties of chalcones.

**Figure 4:**
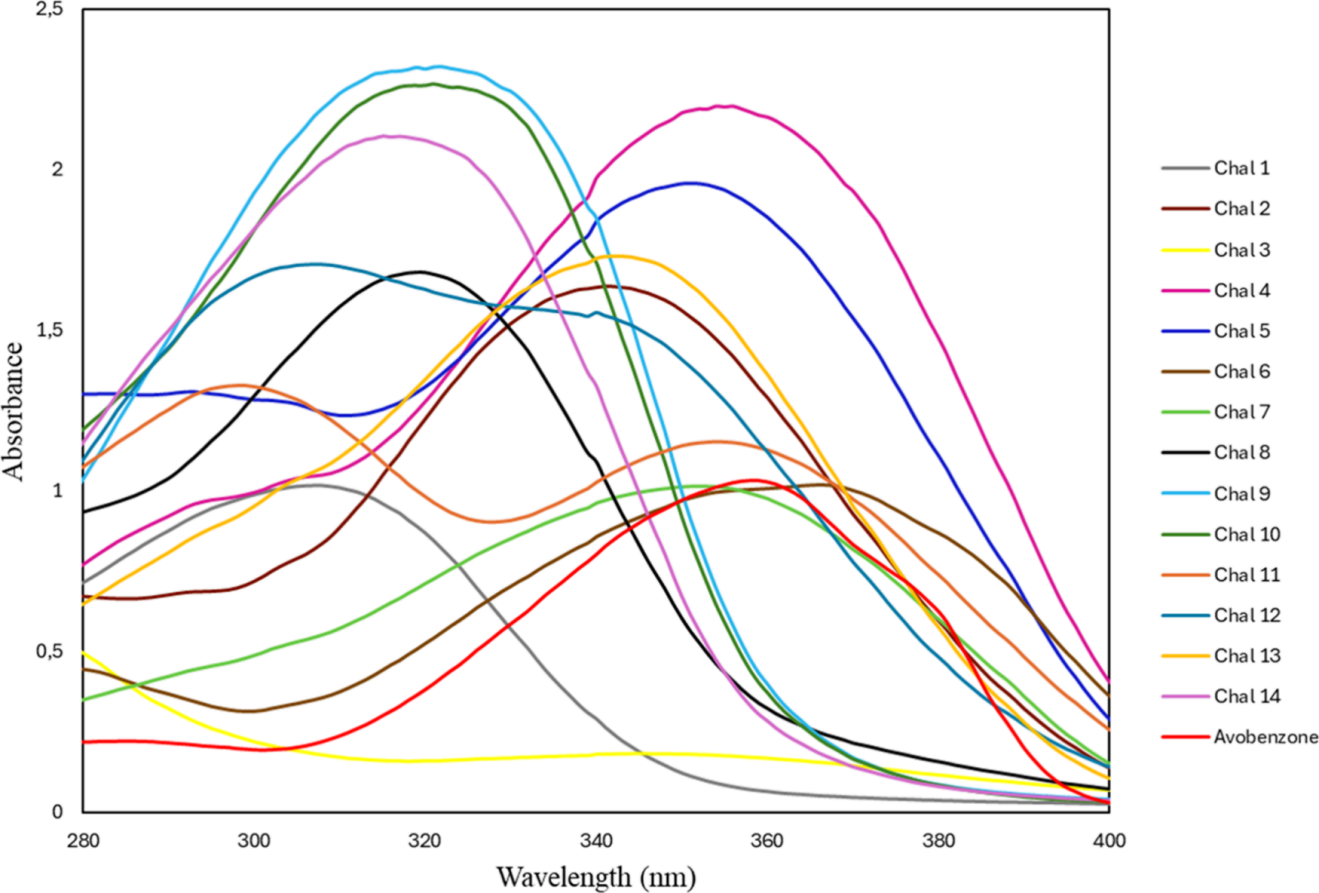
Absorbance profile in the UV region of chalcones and the positive control.

Spectroscopic analysis revealed that the introduction of this substituent induces a significant bathochromic shift in the compounds studied. While unsubstituted chalcone (Chal 1) exhibited a maximum absorption at 307 nm, typical of the basic carbonyl system, methoxy-substituted compounds on ring B (Chal 2–7 and 13) displayed pronounced shifts into the UVA region. Particularly, Chal 2 (λ_max_ = 341 nm) demonstrated a 34 nm shift compared to Chal 1. The methoxy group acts as a strong electron donor, increasing electron density in the π-conjugated system and reducing the HOMO–LUMO energy gap.^22,23^

In addition to the bathochromic shift, the molar extinction coefficient (ε; Table 5) was significantly amplified by the presence of methoxy. Compounds such as Chal 4 (ε = 6.46 × 10^4^ L·mol^–1^·cm^–1^), Chal 5 (ε = 6.09 × 10^4^ L·mol^–1^·cm^–1^), and Chal 13 (ε = 6.20 × 10^4^ L·mol^–1^·cm^–1^) exhibited values superior to that of avobenzone (ε = 3.19 × 10^4^ L·mol^–1^·cm^–1^), indicating remarkably higher absorption efficiency. This parameter is crucial, as it determines the concentration of the filter required in a formulation to achieve the desired sun protection factor (SPF).

**Table 5:** Spectrophotometric Parameters and In Vitro SPF Values of the Chalcones

**Compounds**	**λ**_**máx**_ (nm)	**ε (L·mol** ^**–**^ **^1^** **·cm** ^**–**^ **^1^)**	**λ**_**C**_ (nm)	**SPF** (10 μg·mL^–1^)
Chal 1	307	2.73 × 10^4^	336	3.15
Chal 2	341	5.37 × 10^4^	374	1.89
Chal 3	266	2.08 × 10^4^	352	0.00
Chal 4	354	6.46 × 10^4^	380	0.45
Chal 5	351	6.09 × 10^4^	377	1.10
Chal 6	366	3.21 × 10^4^	387	1.83
Chal 7	352	3.64 × 10^4^	380	2.45
Chal 8	319	4.50 × 10^4^	348	2.17
Chal 9	321	6.22 × 10^4^	350	6.48
Chal 10	323	6.42 × 10^4^	349	5.88
Chal 11	300	4.35 × 10^4^	376	1.56
Chal 12	307	5.84 × 10^4^	367	4.33
Chal 13	343	6.20 × 10^4^	370	2.58
Chal 14	317	6.90 × 10^4^	344	4.03
Avobenzone	360	3.19 × 10^4^	380	1.48

The most relevant parameter for broad-spectrum protection, the critical wavelength (λ_c_) (Table 5), revealed the superior performance of the selected derivatives. Values of λ_c_ ≥ 370 nm are considered indicative of adequate UVA protection. In this respect, Chal 6 (λ_max_ = 366 nm; λ_c_ = 387 nm) emerged as the most promising compound of the series, surpassing the reference standard avobenzone (λ_c_ = 380 nm). This performance can be attributed to a synergistic effect between the para-methoxy group and the ortho-hydroxyl group, which forms an intramolecular hydrogen bond with the carbonyl, conferring molecular rigidity and further extending electronic conjugation.^23^

Complementary in vitro SPF (Table 5) evaluation demonstrated that although the main focus of these derivatives is UVA protection, several compounds also contributed significantly to UVB protection. Chal 9 (SPF = 6.48), 10 (SPF = 5.88), and 14 (SPF = 4.03) exhibited the highest SPF values. It is noteworthy that chalcones containing para-methoxy substitution generally showed higher absorption in the UVA region compared to UVB, confirming their selectivity as specialized UVA filters with appreciable UVB contribution.

The spectroscopic and photoprotective efficacy data position chalcones, particularly Chal 6, as high-performance candidates. The combination of a λ_c_ superior to avobenzone, exceptionally high molar extinction coefficients, and SPF contribution highlights their potential as superior alternatives to currently available UVA filters.

### Photostability

Photostability is an important, yet often overlooked, requirement for organic sunscreens. A filter may exhibit excellent initial absorption properties, but if it degrades rapidly under irradiation, it becomes clinically irrelevant, as its efficacy decreases exponentially during sun exposure and it may also generate photoactive byproducts that are potentially irritating or allergenic.^24^

Initial UV–vis spectrophotometric analysis during 3 h of UVA exposure (Figure 5) revealed that most chalcones underwent a decrease in absorbance, indicative of partial isomerization, while their overall spectral profiles remained largely unchanged. Similarly, Avobenzone displayed a loss of absorbance over time, reflecting its known photoinstability. Chalcone 6 showed an increase in absorbance after just 1 h of exposure, suggesting a different photochemical behavior. These observations prompted further investigation by ^1^H NMR spectroscopy to elucidate the structural changes underlying the observed photophysical responses.

**Figure 5:**
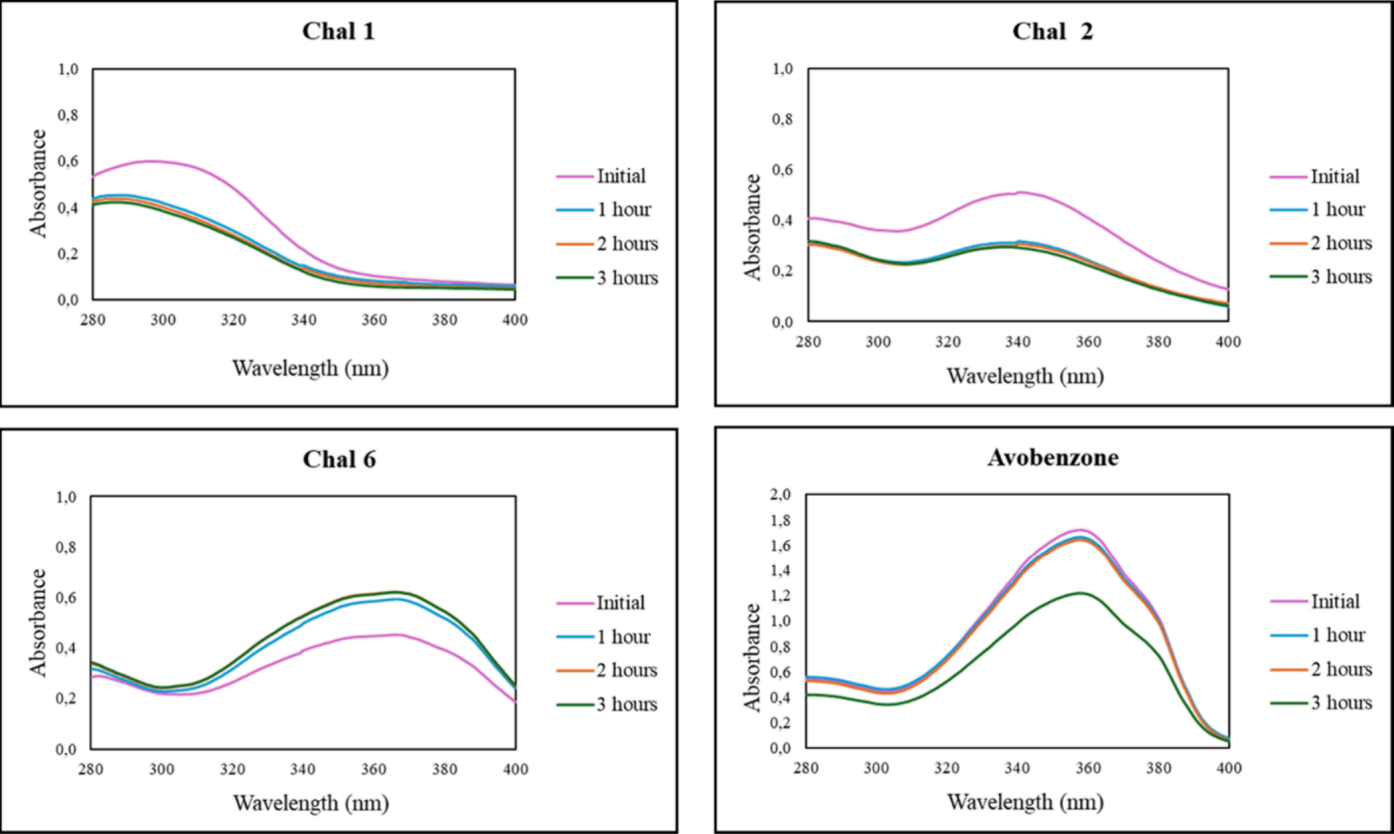
UV–vis spectra of representative compounds (Chal 1, Chal 2, and Chal 6) and avobenzone.

To elucidate the underlying mechanisms, we performed ^1^H NMR studies, which provided structural insights into the compounds (Figures S31–S43). For most of the evaluated chalcones (Chal 1, 2, 4, 7, 8, 9, 10, 12, 13, and 14), partial formation of the *cis* isomer was observed (Figure 6), characterized by the appearance of new signals in the vinylic proton region with reduced coupling constants (*J* = 11–13 Hz), along with a decrease in the intensity of the signals corresponding to the *trans* isomer (*J* = 15–16 Hz). The presence of this *trans–cis* equilibrium is expected for conjugated α,β-unsaturated systems and accounts for the observed reduction in absorbance.

**Figure 6:**
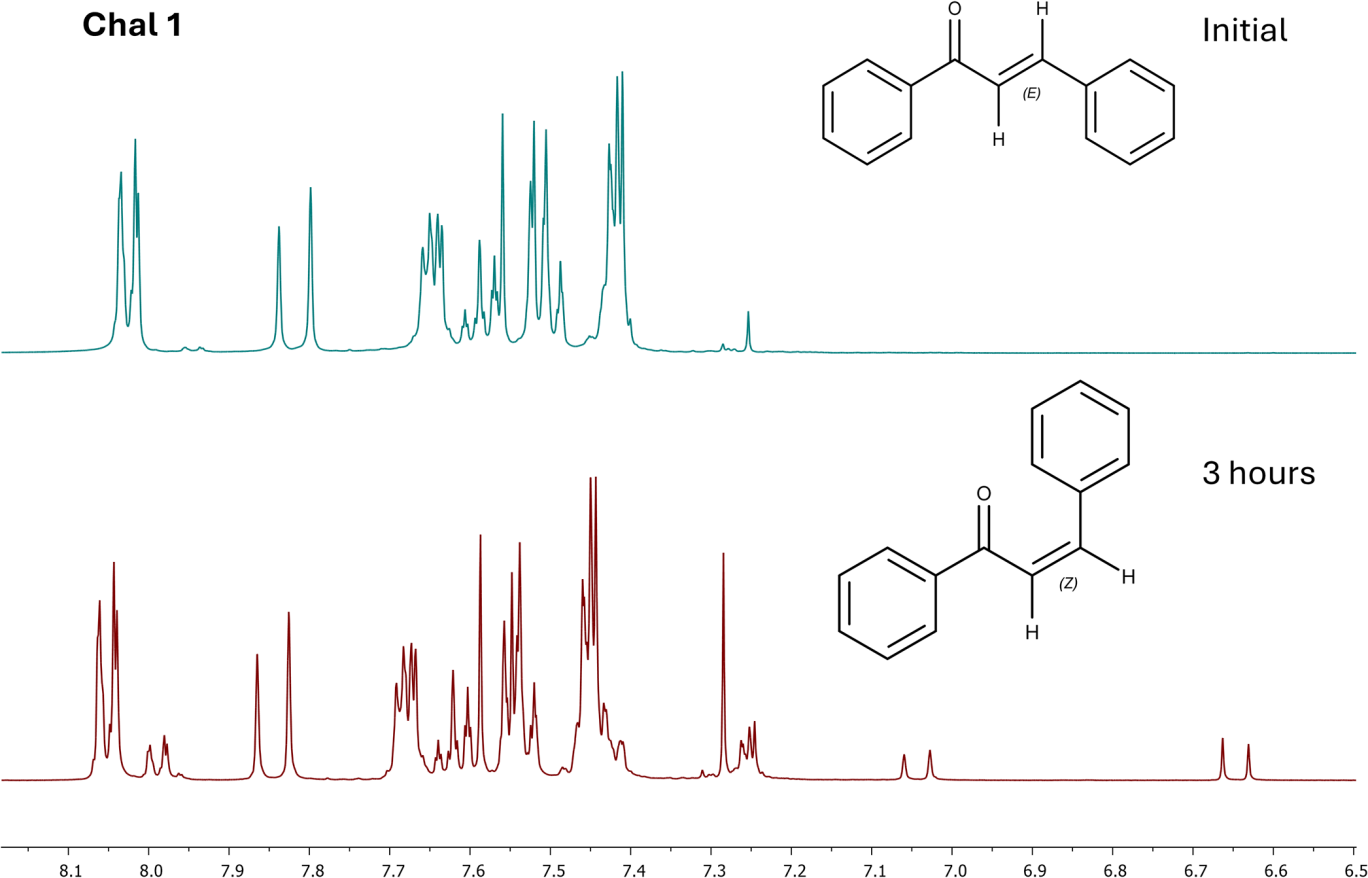
^1^H NMR of Chal 1 before and after exposure to UVA light.

In contrast, the ^1^H NMR spectra of Chal 3, 5, and 11 revealed the appearance of additional signals not attributable to either isomer, indicating the occurrence of advanced photodegradation, possibly involving cyclization, photodimerization, or oxidative cleavage of the molecular structure.

Chal 6, however, exhibited remarkable and highly desirable behavior. After 3 h of irradiation, its ^1^H and ^13^C NMR spectra remained identical to the initial time point, with no evidence of new signals corresponding to the *cis* isomer or degradation products. To investigate whether this stability was maintained under prolonged stress conditions, we performed an exclusive kinetic study, monitoring Chal 6 (Figure 7), 7, 9, 10, and (Figures S44–S46) by ^1^H NMR during 72 h of continuous UVA exposure.

**Figure 7:**
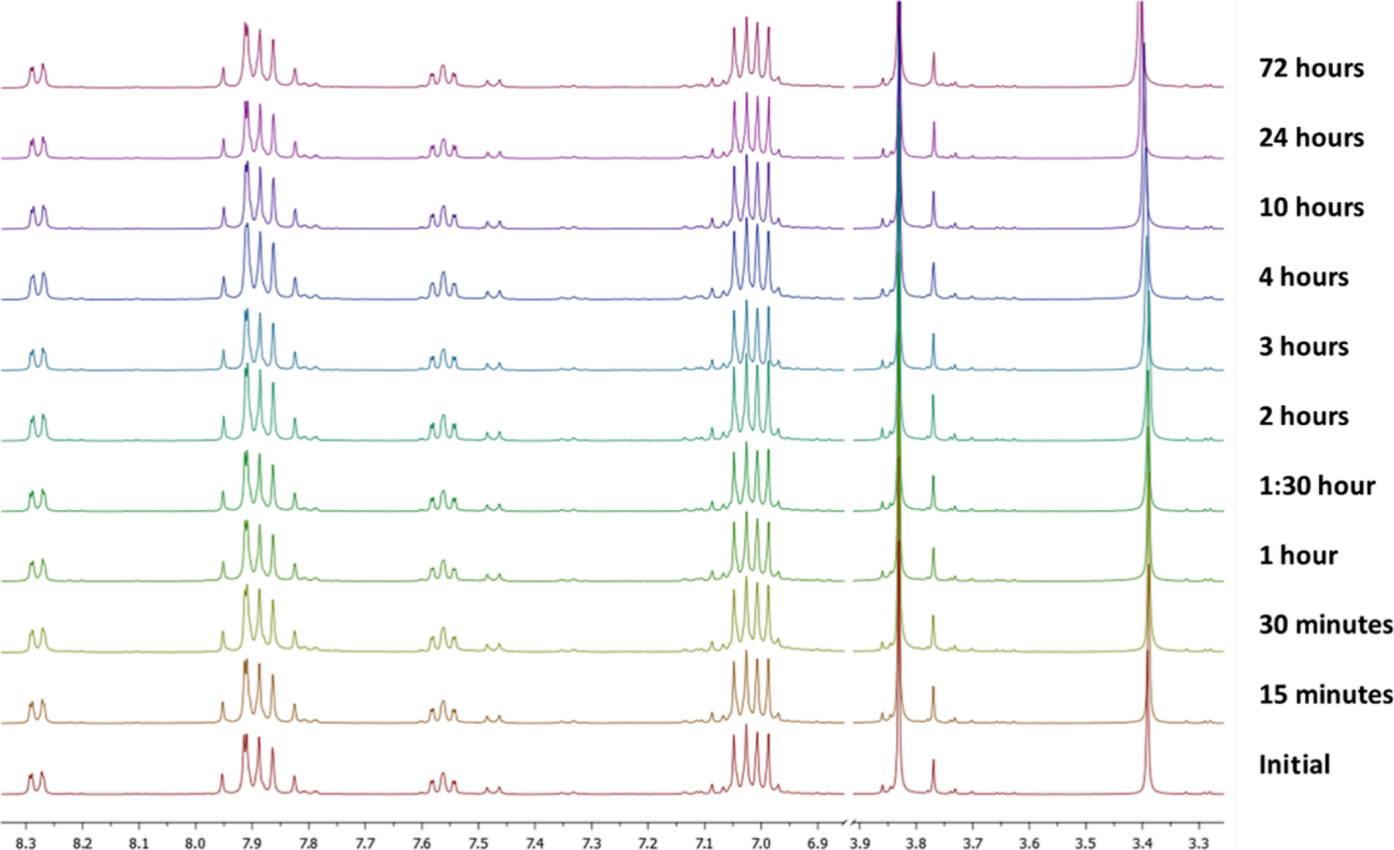
Kinetics of degradation of Chal 6 monitored by ^1^H NMR under UV irradiation.

Quantitative analysis showed that Chal 6 retained its original *trans* configuration after 72 h, which is rarely observed for organic UVA filters. The other chalcones exhibited a gradual decline over time, maintaining approximately 60% of the trans isomer after 72 h (Figure 8).

**Figure 8:**
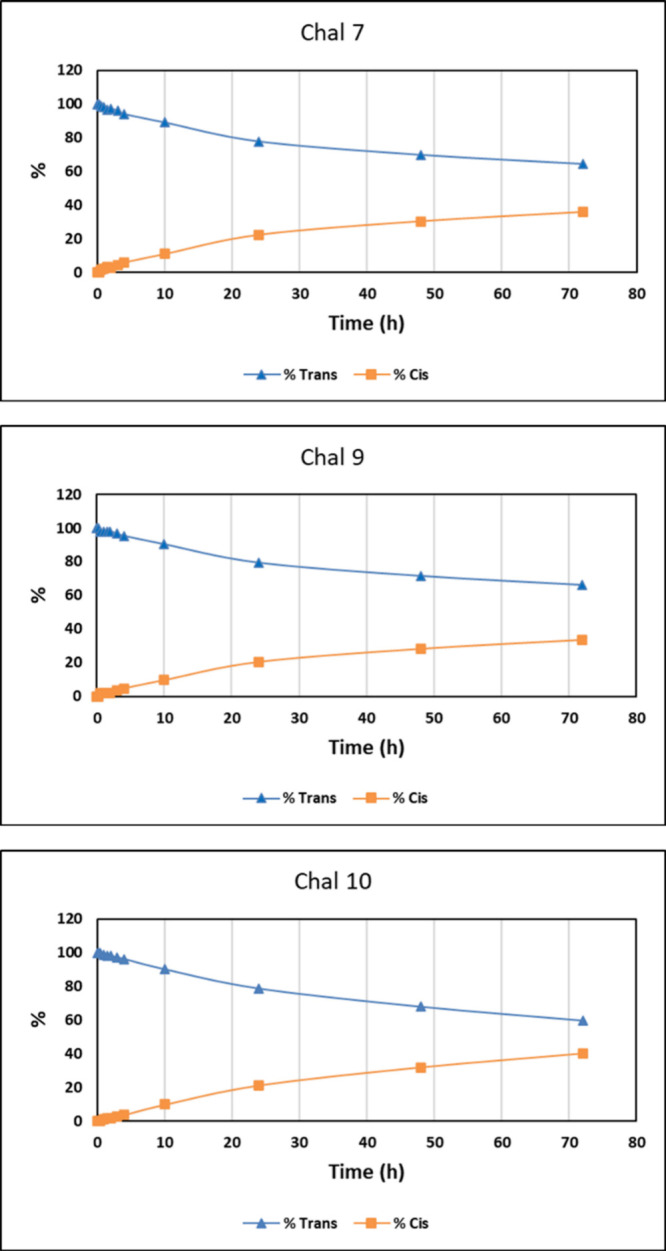
Photoisomerization kinetics (*trans → cis*) of Chalcones 7, 9, and 10 under UV irradiation.

The photostability of Chal 6 is attributed to the combined presence of an ortho-hydroxyl group and a para-methoxy group, which enable the formation of an intramolecular hydrogen bond between the phenolic hydrogen and the carbonyl oxygen. This interaction creates a rigid structure that restricts rotation around the α,β-unsaturated double bond.^25^

While avobenzone, the current standard UVA filter, undergoes significant degradation requiring formulation with stabilizers (octocrylene),^4^ Chal 6 offers superior intrinsic stability. This translates into the promise of consistent and long-lasting UVA protection throughout sun exposure without the need for additives that may complicate formulation or increase the irritant potential of the final product. The combination of broad-spectrum protection (λ_c_ = 387 nm), high absorption efficiency (ε = 3.21 × 10^4^ L·mol^–1^·cm^–1^), and photostability positions Chal 6 not merely as a competitor but as a potential superior substitute for currently available UVA filters.

## Conclusions

The chalcones were effectively synthesized via Claisen–Schmidt condensation, with yields comparable to those reported in the literature, confirming the efficiency of the methodology. Antioxidant activity was investigated through DPPH, FRAP, and electrochemical assays, in which compounds 6, 7, and 12 exhibited the highest free radical scavenging capacity, reducing power, and electron-donating ability, respectively. Rational structural modifications, particularly the introduction of a para-methoxy group, significantly enhanced UV absorption, leading to several derivatives with superior broad-spectrum performance (λ_c_ > 370 nm) compared to avobenzone. Among them, Chal 6 exhibited an outstanding profile, combining high antioxidant potential, broad-spectrum absorption, and remarkable photostability. Its ability to maintain structural integrity and absorption efficiency after 72 h of continuous UVA exposure, as confirmed by ^1^H NMR monitoring, represents a significant improvement over that of conventional UVA filters that typically require stabilizers to prevent photodegradation.

## Materials and Methods

### Synthesis and Characterization

The chalcones were synthesized via aldol condensation between 4-methoxybenzaldehyde, an aldehyde, and different acetophenones. In a 50 mL reaction flask, the corresponding acetophenone (5 mmol) was dissolved in 10 mL of absolute ethanol, followed by the addition of the corresponding aldehyde (5 mmol) and 5 mL of a 50% aqueous NaOH solution. The reaction mixture was stirred at room temperature for 24 h.^26^

At the end of the reaction, the isolation procedures were adjusted according to the derivative. For chalcones 6, 7, 11, and 12, the reaction mixture was acidified with 1 mol L^–1^ HCl to pH ≈ 3, inducing product precipitation, which was subsequently vacuum-filtered and washed with cold water. For the remaining chalcones, precipitation was achieved by direct addition of the reaction mixture to cold water, followed by filtration. All isolated products were recrystallized from ethanol.^26^

**Chal 1** – (2*E*)-1,3-Diphenylprop-2-en-1-one.

Yield: 64.5%. Melting point: 45.6–47.0 °C. ^1^H NMR (400 MHz, CDCl_3_, ppm): 7.82 (d, *J*_*3*_ = 15.73 Hz, 1H, H-β), 7.54 (d, *J*_*3*_ = 15.66 Hz, 1H, H-α), 8.03 (dd, 2H, H-2′/6′), 7.65 (dd, 2H, H-3′/5′), 7.59 (m, 1H, H-4′), 7.50 (m, 2H, H-2/6), 7.42 (m, 3H, H-3/4/5). ^13^C NMR (100 MHz, CDCl_3_, ppm): 190.59 (C = O), 144.87 (C-β), 138.24, 134.92, 132.80, 130.57, 128.99, 128.65, 128.53, 128.47, 122.14 (C-α).

**Chal 2** – (2*E*)-3-(4-Methoxyphenyl)-1-phenylprop-2-en-1-one.

Yield: 74.2%. Melting point: 71.5–72.6 °C. ^1^H NMR (400 MHz, CDCl_3_, ppm): 7.79 (d, *J*_*3*_ = 15.65 Hz, 1H, H-β), 7.42 (d, *J*_*3*_ = 15.64 Hz, 1H, H-α), 8.01 (dt, 2H, H-2′/6′), 7.61 (m, 2H, H-2/6), 7.57 (m, 1H, H-4′), 7.50 (m, 2H, H-3′/5′), 6.94 (m, 2H, H-3/5), 3.86 (s, 3H, OCH_3_). ^13^C NMR (100 MHz, CDCl_3_, ppm): 190.63 (C = O), 161.71, 144.72 (C-β), 138.55, 132.56, 130.24, 128.57, 128.43, 127.66, 119.85 (C-α), 114.45, 55.43.

**Chal 3** – (2*E*)-3-(4-Methoxyphenyl)-1-(2-pyridyl)­prop-2-en-1-one.

Yield: 47.4%. Melting point: 80.2–82.0 °C. ^1^H NMR (400 MHz, CDCl_3_, ppm): 7.92 (d, *J*_*3*_ = 15.98 Hz, 1H, H-β), 8.18 (d, *J*_*3*_ = 15.97 Hz, 1H, H-α), 8.74 (m, 1H, H-3′), 8.18 (m, 1H, H-6′), 7.87 (td, 1H, H-5′), 7.69 (m, 2H, H-2/6), 7.48 (ddd, 1H, H-4′), 6.94 (m, 2H, H-3/5), 3.86 (s, 3H, OCH_3_). ^13^C NMR (100 MHz, CDCl_3_, ppm): 189.43 (C = O), 161.76, 154.52, 148.80, 144.73 (C-β), 136.99, 130.68, 128.00, 126.72, 122.89, 118.55 (C-α), 114.35, 55.42.

**Chal 4** – (2*E*)-1-(Furan-2-yl)-3-(4-methoxyphenyl)­prop-2-en-1-one.

Yield: 53.7%. Melting point: 71.5–74.6 °C. ^1^H NMR (400 MHz, CDCl_3_, δ, ppm): 7.86 (d, *J*_*3*_ = 15.72 Hz, 1H, H-β), 7.34 (d, *J*_*3*_ = 15.83 Hz, 1H, H-α), 7.63 (m, 2H, H-2/6), 7.63 (m, 1H, H-2′), 7.29 (d, 1H, H-4′), 6.94 (m, 2H, H-3/5), 6.59 (dd, 1H, H-3′), 3.86 (s, 3H, OCH_3_). ^13^C NMR (100 MHz, CDCl_3_, ppm): 178.17 (C = O), 161.76, 153.91, 146.27, 143.82 (C-β), 130.34, 127.53, 118.88 (C-α), 117.07, 114.43, 112.45, 55.42.

**Chal 5** – (2*E*)-3-(4-Methoxyphenyl)-1-(2-thienyl)­prop-2-en-1-one.

Yield: 86.0%. Melting point: 75.6–77.6 °C. ^1^H NMR (400 MHz, CDCl_3_, ppm): 7.83 (d, *J*_*3*_ = 15.75 Hz, 1H, H-β), 7.31 (d, *J*_*3*_ = 15.51 Hz, 1H, H-α), 7.86 (d, 1H, H-4′), 7.66 (dd, 1H, H-2′), 7.61 (d, 2H, H-2/6), 7.18 (dd, 1H, H-3′), 6.94 (d, 2H, H-3/5), 3.86 (s, 3H, OCH_3_). ^13^C NMR (100 MHz, CDCl_3_, ppm): 182.09 (C = O), 161.75, 145.81, 143.91 (C-β), 133.50, 131.46, 130.29, 128.17, 127.47, 119.31 (C-α), 114.45, 55.43.

**Chal 6** – (2*E*)-1-(2-Hydroxyphenyl)-3-(4-methoxyphenyl)­prop-2-en-1-one.

Yield: 61.5%. Melting point: 79.2–80.6 °C. ^1^H NMR (400 MHz, CDCl_3_, δ, ppm): 7.90 (d, *J*_*3*_ = 15.40 Hz, 1H, H-β), 7.54 (d, *J*_*3*_ = 15.41 Hz, 1H, H-α), 7.92 (dd, 1H, H-6′), 7.63 (m, 2H, H-2/6), 7.48 (m, 1H, H-4′), 7.02 (m, 1H, H-3′), 6.94 (m, 3H, H-3/5, H-5′), 3.86 (s, 3H, OCH_3_). ^13^C NMR (100 MHz, CDCl_3_, ppm): 193.69 (C = O), 163.58, 162.06, 145.36 (C-β), 136.14, 130.56, 129.54, 127.74, 121.53, 118.75, 118.60, 117.65 (C-α), 114.54, 55.46.

**Chal 7** – (2*E*)-1-(4-Hydroxy-3-methoxyphenyl)-3-(4-methoxyphenyl)­prop-2-en-1-one.

Yield: 39.8%. Melting point: 132.5–135.3 °C. ^1^H NMR (400 MHz, CDCl_3_, ppm): 7.78 (d, *J*_*3*_ = 15.55 Hz, 1H, H-β), 7.44 (d, *J*_*3*_ = 15.55 Hz, 1H, H-α), 7.63 (m, 3H, H-2, H-6, H-2′), 6.97 (dd, 1H, H-3/5), 6.97 (d, 1H, H-5′), 7.63 (m, 1H, H-6′), 3.99 (s, 3H, OCH_3_), 3.86 (s, 3H, OCH_3_). ^13^C NMR (100 MHz, CDCl_3_, ppm): 188.62 (C = O), 161.54, 150.19, 146.84, 143.83 (C-β), 131.32, 130.12, 127.84, 123.51 (C-α), 119.37, 114.40, 113.76, 110.50, 56.15, 55.42

**Chal 8** – (2*E*)-3-Phenyl-1-(2-pyridyl)­prop-2-en-1-one.

Yield: 37.5%. Melting point: 69.3–69.5 °C. ^1^H NMR (400 MHz, CDCl_3_, ppm): 7.94 (d, *J* = 16.05 Hz, 1H, H-β), 8.30 (d, *J*_*3*_ = 16.06 Hz, 1H, H-α), 7.73 (m, 2H, H-2/6), 7.42 (m, 2H, H-3/5), 7.42 (m, 1H, H-4), 8.74 (m, 1H, H-3′), 7.49 (ddd, 1H, H-4′), 7.88 (td, 1H, H-5′), 8.19 (m, 1H, H-6′). ^13^C NMR (100 MHz, CDCl_3_, ppm): 189.54 (C = O), 154.28, 148.88, 144.82 (C-β), 137.03, 135.20, 130.57, 128.87, 126.89, 122.95, 120.92 (C-α).

**Chal 9** – (2*E*)-1-(Furan-2-yl)-3-phenylprop-2-en-1-one.

Yield: 31.8%. Melting point: 85.6–86.3 °C. ^1^H NMR (400 MHz, CDCl_3_, ppm): 7.91 (d, *J*_*3*_ = 15.80 Hz, 1H, H-β), 7.48 (d, *J*_*3*_ = 15.82 Hz, 1H, H-α), 7.68 (m, 2H, H-2/6), 7.44 (m, 2H, H-3/5), 7.44 (m, 1H, H-4), 7.68 (m, 1H, H-2′), 6.63 (dd, 1H, H-3′), 7.36 (d, 1H, H-4′). ^13^C NMR (100 MHz, CDCl_3_, ppm): 178.05 (C = O), 153.78, 146.47, 144.00 (C-β), 134.80, 130.59, 128.95, 128.53, 121.25 (C-α), 117.42, 112.52.

**Chal 10** – (2*E*)-3-Phenyl-1-(2-thienyl)­prop-2-en-1-one.

Yield: 39.6%. Melting point: 79.6–80.2 °C. ^1^H NMR (400 MHz, CDCl_3_, ppm): 7.85 (d, *J*_*3*_ = 15.73 Hz, 1H, H-β), 7.42 (d, *J*_*3*_ = 15.60 Hz, 1H, H-α), 7.64 (m, 2H, H-2/6), 7.43 (m, 2H, H-3/5), 7.43 (m, 1H, H-4), 7.69 (dd, 1H, H-2′), 7.19 (dd, 1H, H-3′), 7.87 (d, 1H, H-4′). ^13^C NMR (100 MHz, CDCl_3_, ppm): 182.07 (C = O), 145.54, 144.12 (C-β), 134.75, 133.88, 131.81, 130.61, 128.99, 128.50, 128.25, 121.68 (C-α).

**Chal 11** – (2*E*)-1-(2-Hydroxyphenyl)-3-phenylprop-2-en-1-one.

Yield: 33.2%. Melting point: 87.3–89.0 °C. ^1^H NMR (400 MHz, CDCl_3_, ppm): 8.23 (d, *J*_*3*_ = 15.88 Hz, 1H, H-β), 7.74 (d, *J*_*3*_ = 15.88 Hz, 1H, H-α), 8.07 (m, 2H, H-2/6), 7.53 (m, 2H, H-3/5), 7.61 (m, 1H, H-4), 6.98 (m, 1H, H-3′), 7.61 (m, 1H, H-4′), 6.98 (m, 1H, H-5′), 7.30 (m, 1H, H-6′). ^13^C NMR (100 MHz, CDCl_3_, ppm): 192.14 (C = O), 155.96, 141.17 (C-β), 138.34, 132.79, 131.85, 129.45, 128.69, 128.61, 122.73 (C-α), 122.25, 120.83, 116.68.

**Chal 12** – (2*E*)-1-(4-Hydroxy-3-methoxyphenyl)-3-phenylprop-2-en-1-one.

Yield: 35.7%. Melting point: 46.6–50.0 °C. ^1^H NMR (400 MHz, CDCl_3_, ppm): 7.81 (d, *J*_*3*_ = 15.64 Hz, 1H, H-β), 7.55 (d, *J*_*3*_ = 15.64 Hz, 1H, H-α), 7.66 (m, 2H, H-2/6), 7.43 (m, 3H, H-3/4/5), 7.66 (m, 1H, H-2′), 7.00 (d, 1H, H-5′), 7.66 (m, 1H, H-6′), 4.00 (s, 3H, OCH_3_). ^13^C NMR (100 MHz, CDCl_3_, ppm): 188.56 (C = O), 150.37, 146.88, 143.97 (C-β), 135.11, 131.08, 130.35, 128.94, 128.37, 123.71, 121.70 (C-α), 113.79, 110.49, 56.17.

**Chal 13** – (2*E*)-1,3-Bis­(4-methoxyphenyl)­prop-2-en-1-one.

Yield: 69.7%. Melting point: 102.1–103.7 °C. ^1^H NMR (400 MHz, CDCl_3_, ppm): 7.80 (d, *J*_*3*_ = 15.58 Hz, 1H, H-β), 7.46 (d, *J*_*3*_ = 15.57 Hz, 1H, H-α), 7.62 (d, 2H, H-2/6), 6.99 (m, 2H, H-3/5), 3.87 (s, 3H, OCH_3_), 8.06 (d, 2H, H-2′/6′), 6.99 (m, 2H, H-3′/5′), 3.91 (s, 3H, OCH_3_). ^13^C NMR (100 MHz, CDCl_3_, ppm): 188.80 (C = O), 163.29, 161.53, 143.82 (C-β), 131.40, 130.70, 130.10, 127.85, 119.62 (C-α), 114.80, 113.80, 55.48, 55.41.

**Chal 14** – (2*E*)-1-(4-Methoxyphenyl)-3-phenylprop-2-en-1-one.

Yield: 94.3%. Melting point: 104.6–108.6 °C. ^1^H NMR (400 MHz, CDCl_3_, δ, ppm): 7.80 (d, *J*_*3*_ = 15.66 Hz, 1H, H-β), 7.55 (d, *J*_*3*_ = 15.66 Hz, 1H, H-α), 7.64 (m, 2H, H-2/6), 7.43 (m, 3H, H-3/4/5), 8.04 (m, 2H, H-2′/6′), 6.99 (m, 2H, H-3′/5′), 3.89 (s, 3H, OCH_3_). ^13^C NMR (100 MHz, CDCl_3_, ppm): 188.76 (C = O), 163.46, 143.99 (C-β), 135.12, 131.13, 130.84, 130.34, 128.94, 128.37, 121.93 (C-α), 113.87, 55.51.

The synthesized compounds were characterized by melting point, determined using a digital melting point apparatus and by nuclear magnetic resonance (NMR) spectroscopy. The ^1^H and ^13^C NMR spectra were recorded on a Bruker Avance III spectrometer (400 MHz for ^1^H and 100 MHz for ^13^C) using CDCl_3_ as the solvent and tetramethylsilane (TMS) as the internal standard.

### Antioxidant Activity

#### DPPH Radical Scavenging Assay

The antioxidant activity of the chalcones was assessed via DPPH radical scavenging, adapted from Sachett et al.^27^ A 0.2 mmol·L^–1^ DPPH solution in ethanol was used. In 96-well plates, 100 μL of chalcone solutions (6.25–100 μmol·mL^–1^, in triplicate) were mixed with 100 μL of DPPH solution. Plates were incubated in the dark for 30 min, and the absorbance was read at 517 nm. Radical inhibition was calculated as described in eq 3.

$$I\;(\%)\;=\;\left(\frac{1-(A{bs}_{\mathrm{sample}}\;-\;{Abs}_{\mathrm{blank}})}{{Abs}_{\mathrm{DPPH}}}\right)\;\times \;100$$ 3

The data obtained were used to determine the IC_5__0_ from the regression curve between the sample concentration and percentage inhibition. Ascorbic acid was used as a positive control.

#### FRAP Assay

The antioxidant capacity of the chalcones was evaluated using the assay, following a methodology adapted from Sachett et al.^28^ The following solutions were prepared: acetate buffer (300 mmol·L^–1^, pH 3.6), obtained by dissolving sodium acetate in ultrapure water and adjusting the pH with glacial acetic acid; TPTZ solution (10 mmol·L^–1^), prepared by dissolving TPTZ in 40 mmol·L^–1^ HCl; and FeCl_3_·6H_2_O solution (20 mmol·L^–1^), freshly prepared on the day of analysis. The FRAP working solution was prepared by mixing the solutions in a 10:1:1 ratio (acetate buffer:TPTZ:FeCl_3_·6H_2_O) and kept protected from light until use.

For the standard curve, FeSO_4_·7H_2_O solutions (100–2000 μmol·L^–1^) were added to the FRAP reagent. Chalcone samples were prepared in microtubes with ultrapure water and FRAP working solution, following the same procedure as that for the standards. Solutions were incubated at 37 °C for 15 min and then transferred in triplicate to 96-well plates, and absorbance was measured at 593 nm using an ELISA plate reader. Antioxidant capacity was determined by interpolation from the standard curve and expressed as mmol Fe^2^^+^ equivalents per mmol of sample.

#### Electrochemical Assay

Electrochemical studies were conducted at room temperature using a Metrohm Autolab potentiostat/galvanostat (PGSTAT204) controlled by NOVA 2.1.7 software. Measurements were performed using a standard three-electrode system: a glassy carbon electrode (GCE) as the working electrode, a Ag/AgCl (3 mol·L^–1^) electrode as the reference, and a platinum wire as the counter electrode.

The electrolyte solution consisted of 0.04 mol·L^–1^ Britton-Robinson (BR) buffer at pH levels of 2, 4, 7, and 10, prepared from 0.04 mol·L^–1^ phosphoric acid, 0.04 mol·L^–1^ boric acid, and 0.04 mol·L^–1^ acetic acid, with pH adjustments made as needed using 0.5 mol·L^–1^ NaOH. The chalcones were dissolved in a 1:1 (v/v) mixture of ethanol and BR buffer to a final concentration of 1.0 × 10^–3^ mol·L^–1^.

Cyclic voltammograms were recorded in the potential range of – 0.2 to +1.4 V (vs Ag/AgCl) at a scan rate of 0.1 V s^–1^. Prior to measurements, the GCE was polished with 0.3 μm alumina slurry on a polishing pad and cleaned in an ultrasonic ethanol bath for 5 min.^26^

### Photoprotective Activity

#### Determination of the SPF

The SPF of the chalcones was determined using the spectrophotometric method described by Mansur et al.^29^ Initially, a gel was prepared containing 3% (w/w) Pemulen TR-1 and 2% (w/w) propylene glycol, homogenized in distilled water up to 150 g for 1 h using a mechanical stirrer. The formulation pH was adjusted to 6, and 0.2% of the preservative Cosmoguard was added.

The chalcones were incorporated into the gel at a ratio of 10 mg/gram of gel and subsequently diluted in 70% ethanol to a final concentration of 10 μg·mL^–1^. Avobenzone at the same concentration was used as the standard control. Absorbance readings were recorded at 5 nm intervals over the 290–320 nm spectrum.^24^ The absorbance values were then applied to eq 4 for SPF calculation.^30^

$$SPF\;=\;FC\sum \frac{320}{290}\;EE(\lambda ).I(\lambda ).Abs(\lambda )$$ 4

where *FC* = 10 (constant), *EE* is the erythemal effect, *I* is the relative intensity of sunlight, and *Abs* is the sample absorbance.

The values of *EE* × *I* are constants experimentally determined by Mansur et al., as shown in Table 6:

**Table 6:** Erythemal Effect (*EE*) × Radiation Intensity (*I*) versus Wavelength (λ)

λ (nm)	EE (λ) × I (λ)
290	0.0150
295	0.0817
300	0.2874
305	0.3278
310	0.1864
315	0.0839
320	0.0180

### Photostability

The photostability of the chalcones was evaluated by using a light chamber equipped with a 365 nm UV lamp, simulating solar exposure. Initially, sample solutions were prepared in 70% ethanol at 10 mg·mL^–1^ and incorporated into a 3% Pemulen gel at 10 mg·g^–1^. The vials containing the solutions were exposed to UV radiation for a total of 3 h, with spectrophotometric measurements taken every hour. Sample absorbance was recorded using a UV–Vis spectrophotometer over the 200–400 nm wavelength range.^31^

The evaluation was performed by monitoring changes in absorbance over time, with results expressed as absorbance versus time plots, allowing visualization of the degradation rate of the tested photoprotective compounds.^30^ For some chalcones, ^1^H NMR monitoring was also conducted for up to 72 h, enabling observation of structural changes during UV exposure.

## Supplementary Material



## Notes

^1^H NMR spectrum (CDCl3, 400 MHz) of Chal 1; ^13^C NMR spectrum (CDCl3, 100 MHz) of Chal 1; ^1^H NMR spectrum (CDCl3, 400 MHz) of Chal 2; ^13^C NMR spectrum (CDCl3, 100 MHz) of Chal 2; ^1^H NMR spectrum (CDCl3, 400 MHz) of Chal 3; ^13^C NMR spectrum (CDCl3, 100 MHz) of Chal 3; ^1^H NMR spectrum (CDCl3, 400 MHz) of Chal 4; ^13^C NMR spectrum (CDCl3, 100 MHz) of Chal 4; ^1^H NMR spectrum (CDCl3, 400 MHz) of Chal 5; ^13^C NMR spectrum (CDCl3, 100 MHz) of Chal 5; ^1^H NMR spectrum (CDCl3, 400 MHz) of Chal 6; ^13^C NMR spectrum (CDCl3, 100 MHz) of Chal 6; ^1^H NMR spectrum (CDCl3, 400 MHz) of Chal 7; ^13^C NMR spectrum (CDCl3, 100 MHz) of Chal 7; ^1^H NMR spectrum (CDCl3, 400 MHz) of Chal 8; ^13^C NMR spectrum (CDCl3, 100 MHz) of Chal 8; ^1^H NMR spectrum (CDCl3, 400 MHz) of Chal 9; ^13^C NMR spectrum (CDCl3, 100 MHz) of Chal 9; ^1^H NMR spectrum (CDCl3, 400 MHz) of Chal 10; ^13^C NMR spectrum (CDCl3, 100 MHz) of Chal 10; ^1^H NMR spectrum (CDCl3, 400 MHz) of Chal 11; ^13^C NMR spectrum (CDCl3, 100 MHz) of Chal 11; ^1^H NMR spectrum (CDCl3, 400 MHz) of Chal 12; ^13^C NMR spectrum (CDCl3, 100 MHz) of Chal 12; ^1^H NMR spectrum (CDCl3, 400 MHz) of Chal 13; ^13^C NMR spectrum (CDCl3, 100 MHz) of Chal 13; ^1^H NMR spectrum (CDCl3, 400 MHz) of Chal 14; ^13^C NMR spectrum (CDCl3, 100 MHz) of Chal 14; cyclic voltammograms of Chal 1 in BR buffer 0.04 M pH 2–10 and ethanol 1:1 (v/v), potential range −0.2 to 1.4 V, scan rate 0.1 V s^–1^; relationships between pH vs *E* obtained from cyclic voltammograms; ^1^H NMR of Chal 2 before and after UVA exposure; ^1^H NMR of Chal 3 before and after UVA exposure; ^1^H NMR of Chal 4 before and after UVA exposure; ^1^H NMR of Chal 5 before and after UVA exposure; ^1^H NMR of Chal 6 before and after UVA exposure; ^1^H NMR of Chal 7 before and after UVA exposure; ^1^H NMR of Chal 8 before and after UVA exposure; ^1^H NMR of Chal 9 before and after UVA exposure; ^1^H NMR of Chal 10 before and after UVA exposure; ^1^H NMR of Chal 11 before and after UVA exposure; ^1^H NMR of Chal 12 before and after UVA exposure; ^1^H NMR of Chal 13 before and after UVA exposure; ^1^H NMR of Chal 14 before and after UVA exposure; kinetics of degradation of Chal 7 monitored by ^1^H NMR under UV irradiation; kinetics of degradation of Chal 9 monitored by ^1^H NMR under UV irradiation; and kinetics of degradation of Chal 10 monitored by ^1^H NMR under UV irradiation (PDF)
