# Supplementary material for: Chalcone Derivatives: Antioxidant Activity, Photoprotective Properties, and Stability under UV Irradiation
Source: ACS Omega. 2025 Dec 15;10(51):63061–71. doi: 10.1021/acsomega.5c08934 (PMC12756833; doi:10.1021/acsomega.5c08934)

Supplementary Material

Chalcone Derivatives: Antioxidant Activity, Photoprotective Properties and Stability under UV Irradiation

Raphaela P. Guaringue[a], Vinícius M. Schaffka[a], Larissa Kozan[a], André L. Kerek[a], Eduardo Protachevicz[a], André V. Bassani[a], Christiana A. Pessoa[a], Patrícia M. Campos[b], Guilherme A. Camargo[c], Barbara C. Fiorin[a*]

[a] *Chemistry Department, State University of Ponta Grossa, Av. Carlos Cavalcanti, 4748, 84030-900, Ponta Grossa, Brazil*
[b] *Department of Pharmaceutical Sciences, State University of Ponta Grossa, Av. Carlos Cavalcanti, 4748, 84030-900, Ponta Grossa, Brazil.*
[c] *Department of Pharmacy, Federal University of Paraná, Av. Prefeito Lothário Meissner, 632, 80210-170, Curitiba, Brazil.*

E-mail addresses: bcfiorin@uepg.br*

Figure S1. ^{1}H NMR spectrum ($CDCl_3$, 400 MHz) of Chal 1.

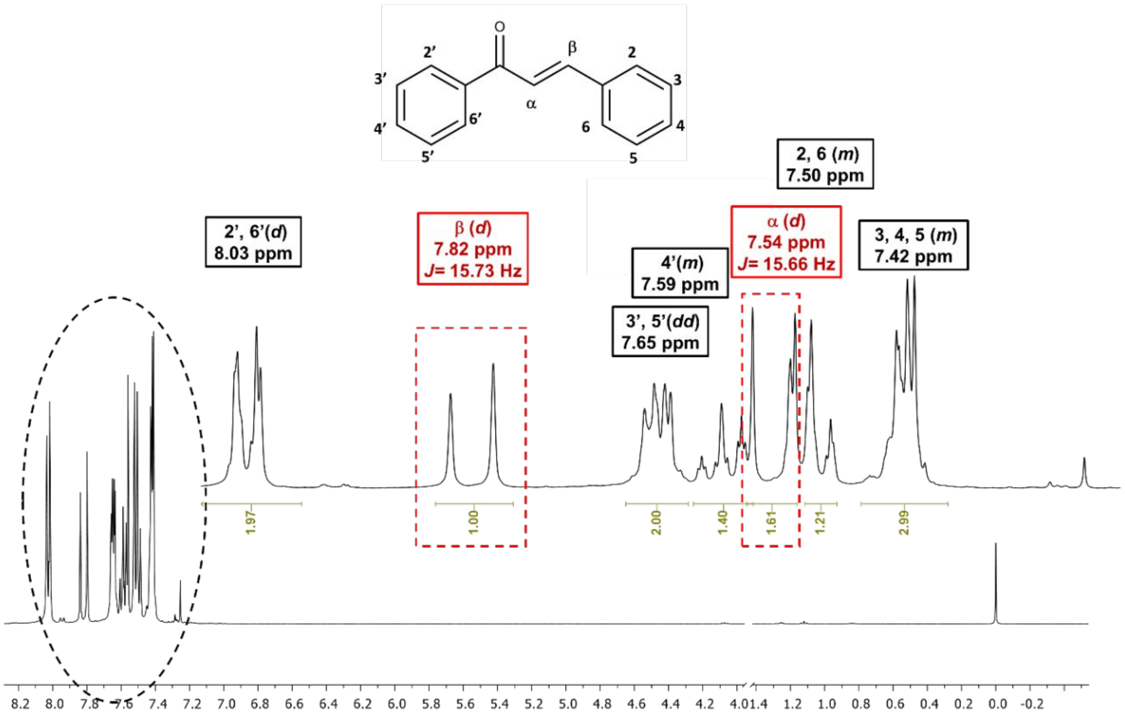

Figure S2. ^{13}C NMR spectrum ($CDCl_3$, 100 MHz) of Chal 1.

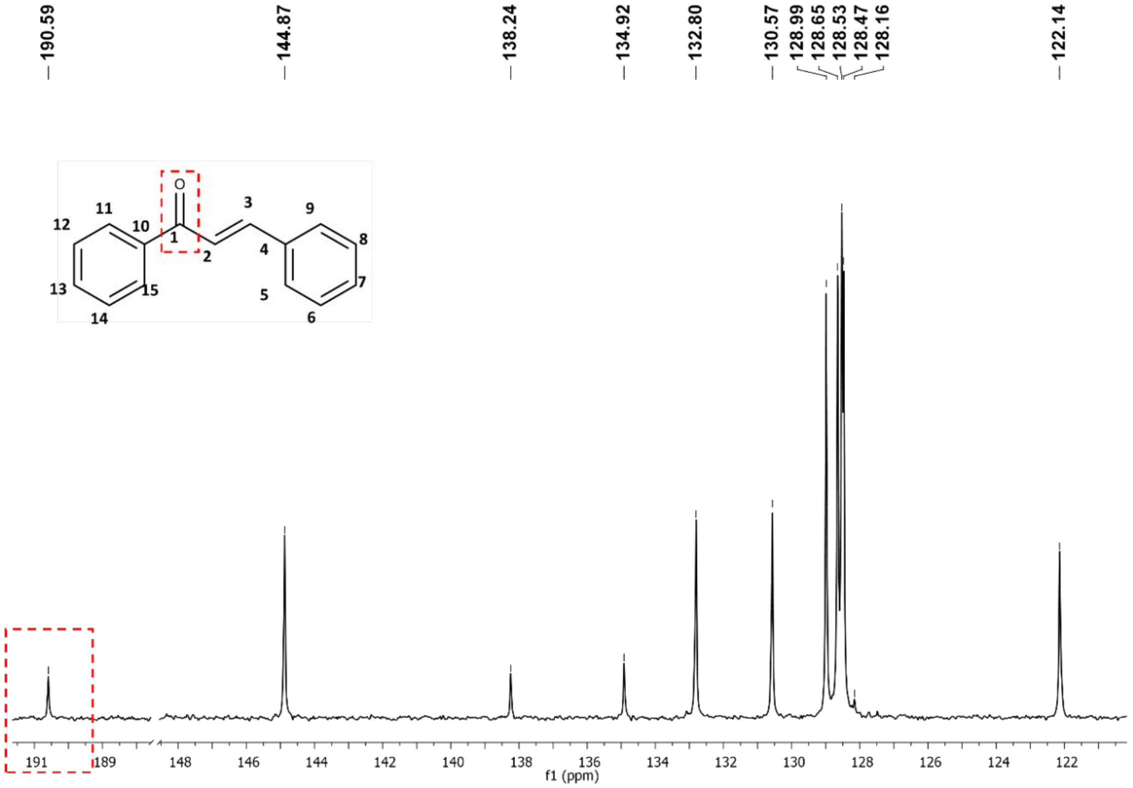

Figure S3. ^{1}H NMR spectrum ($CDCl_3$, 400 MHz) of Chal 2.

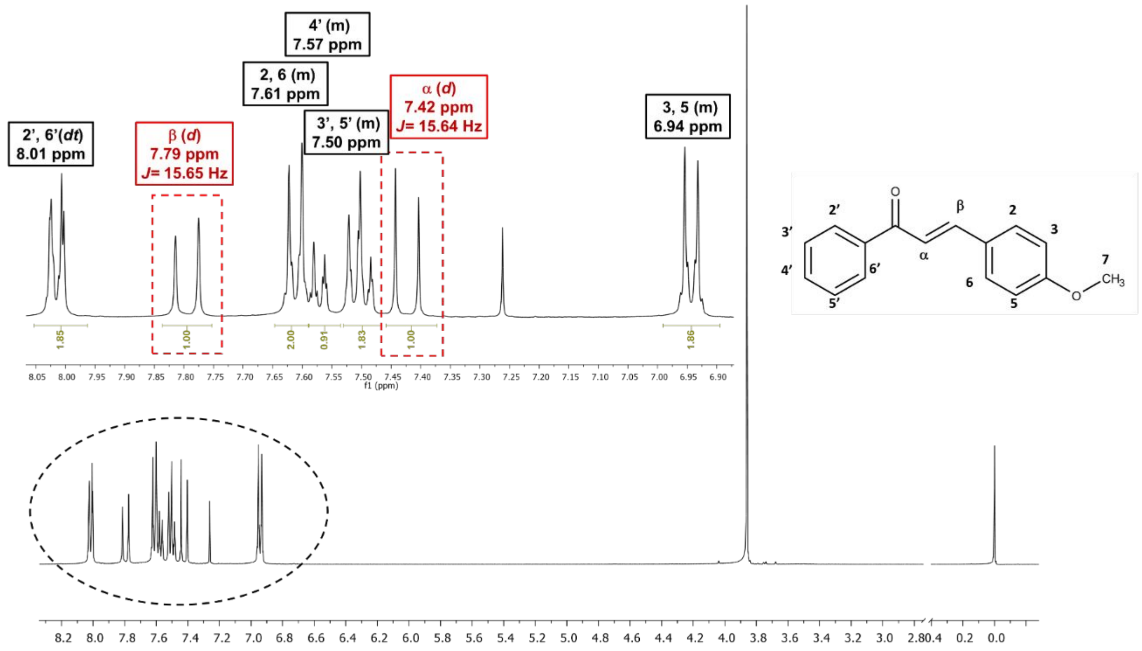

Figure S4. ^{13}C NMR spectrum ($CDCl_3$, 100 MHz) of Chal 2.

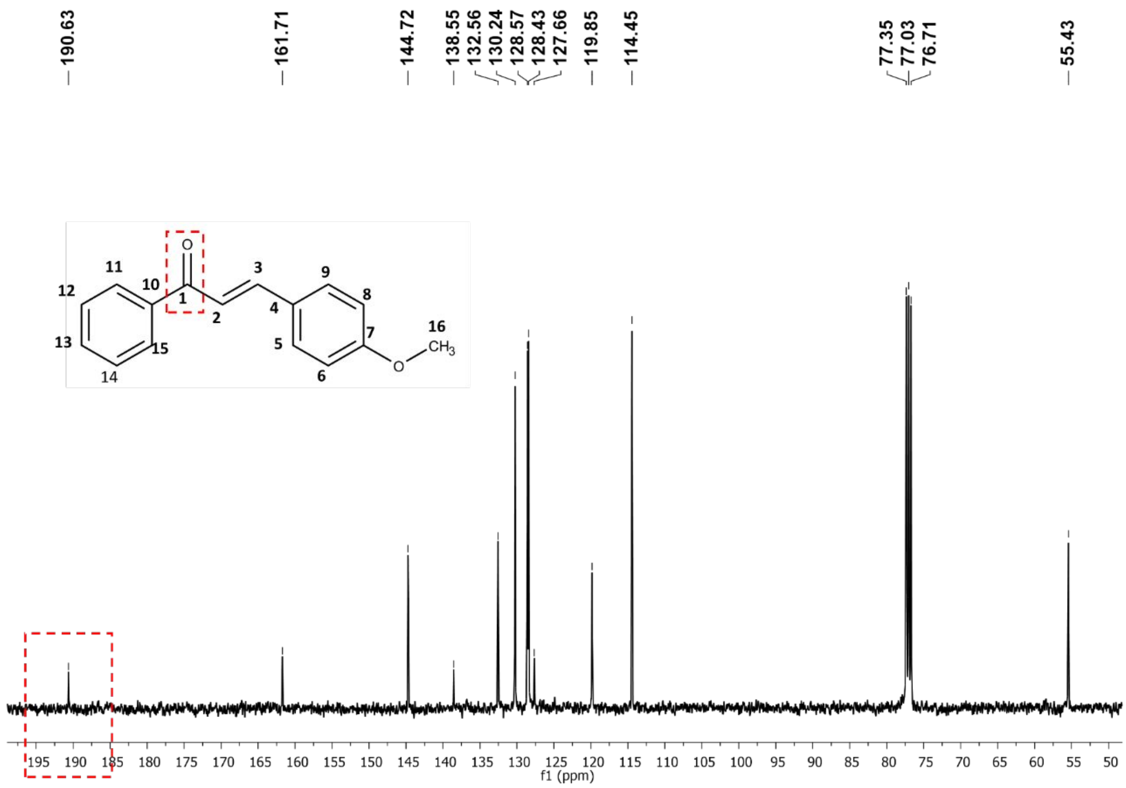

Figure S5. ^{1}H NMR spectrum ($CDCl_3$, 400 MHz) of Chal 3.

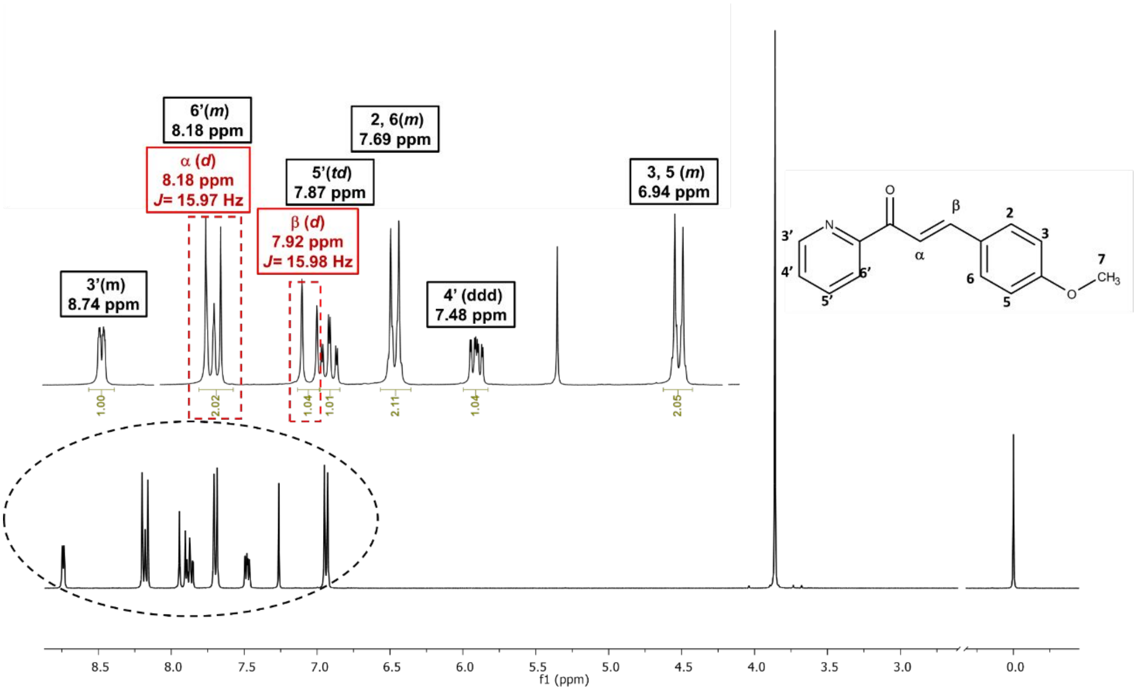

Figure S6. ^{13}C NMR spectrum ($CDCl_3$, 100 MHz) of Chal 3.

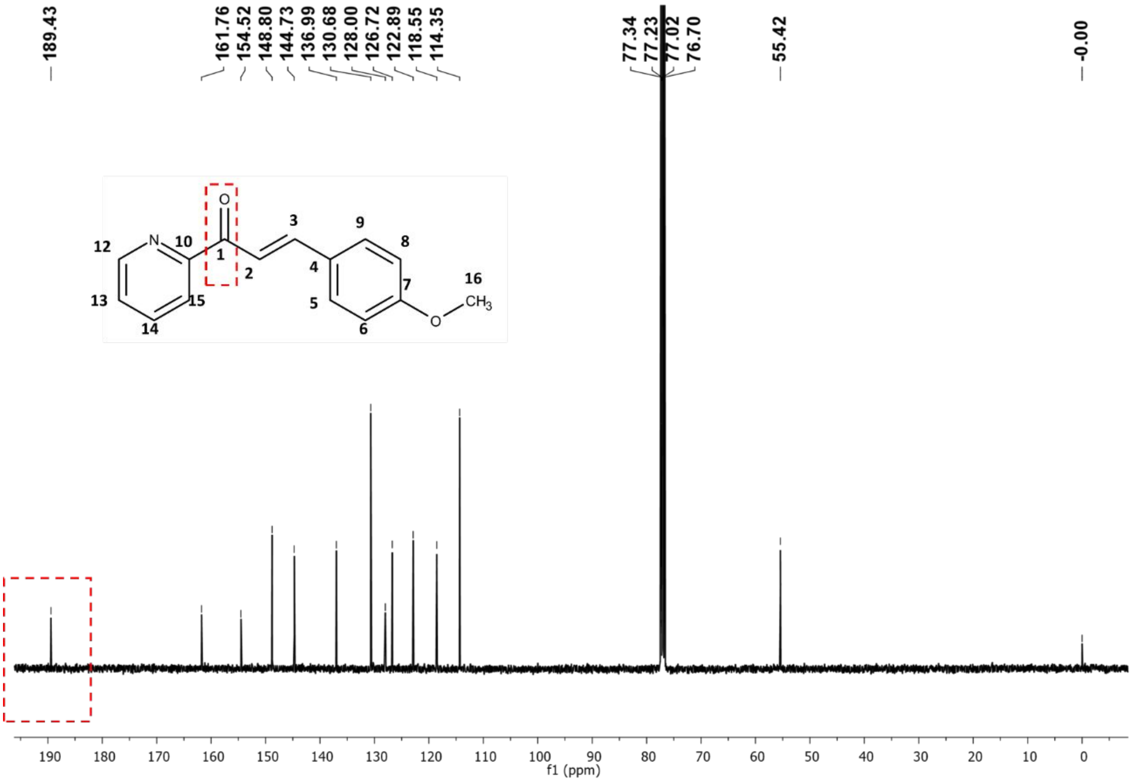

Figure S7. ^1H NMR spectrum ($CDCl_3$, 400 MHz) of Chal 4.

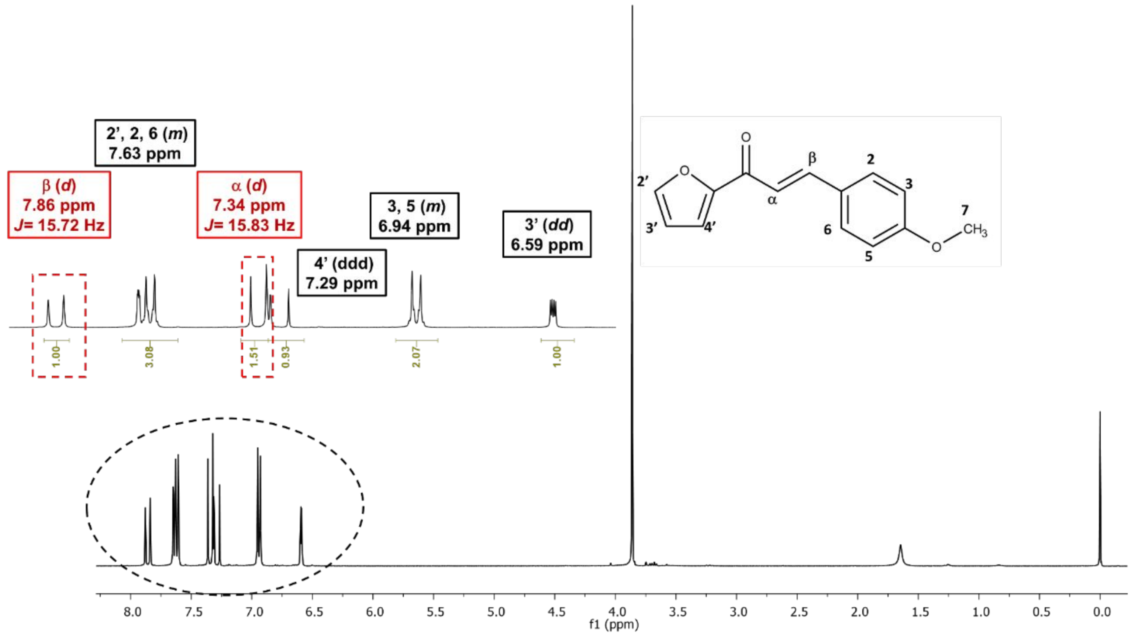

Figure S8. ^{13}C NMR spectrum ($CDCl_3$, 100 MHz) of Chal 4.

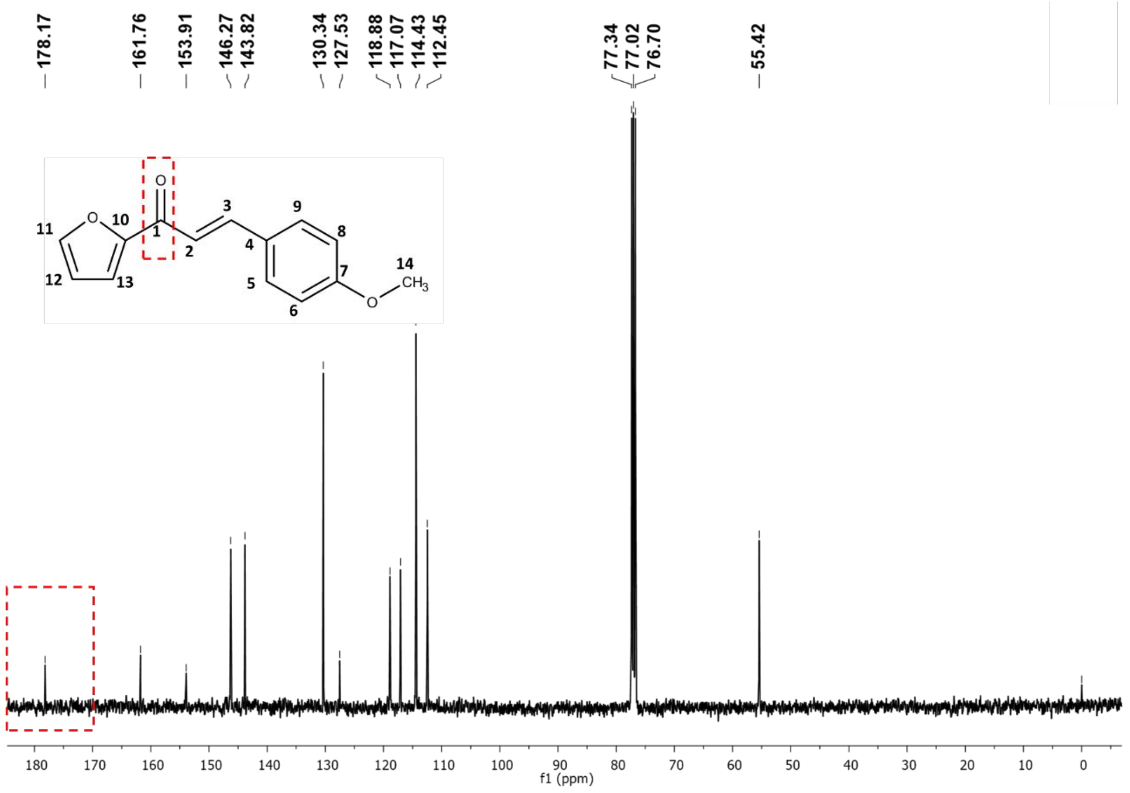

Figure S9. ^{1}H NMR spectrum ($CDCl_3$, 400 MHz) of Chal 5.

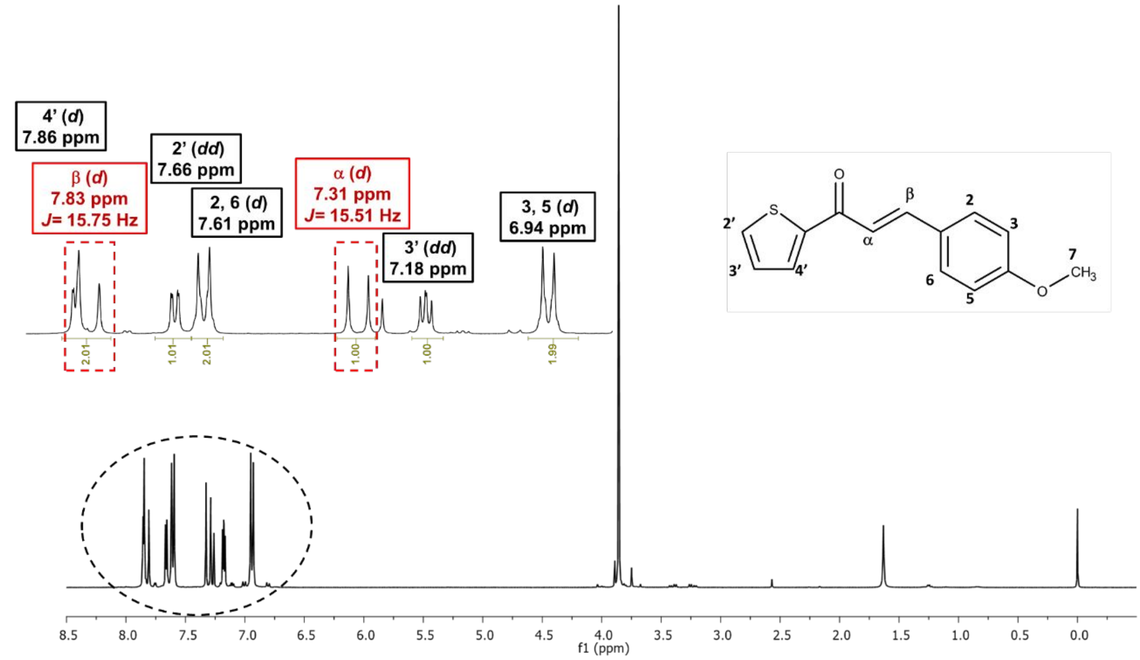

Figure S10. ^{13}C NMR spectrum ($CDCl_3$, 100 MHz) of Chal 5.

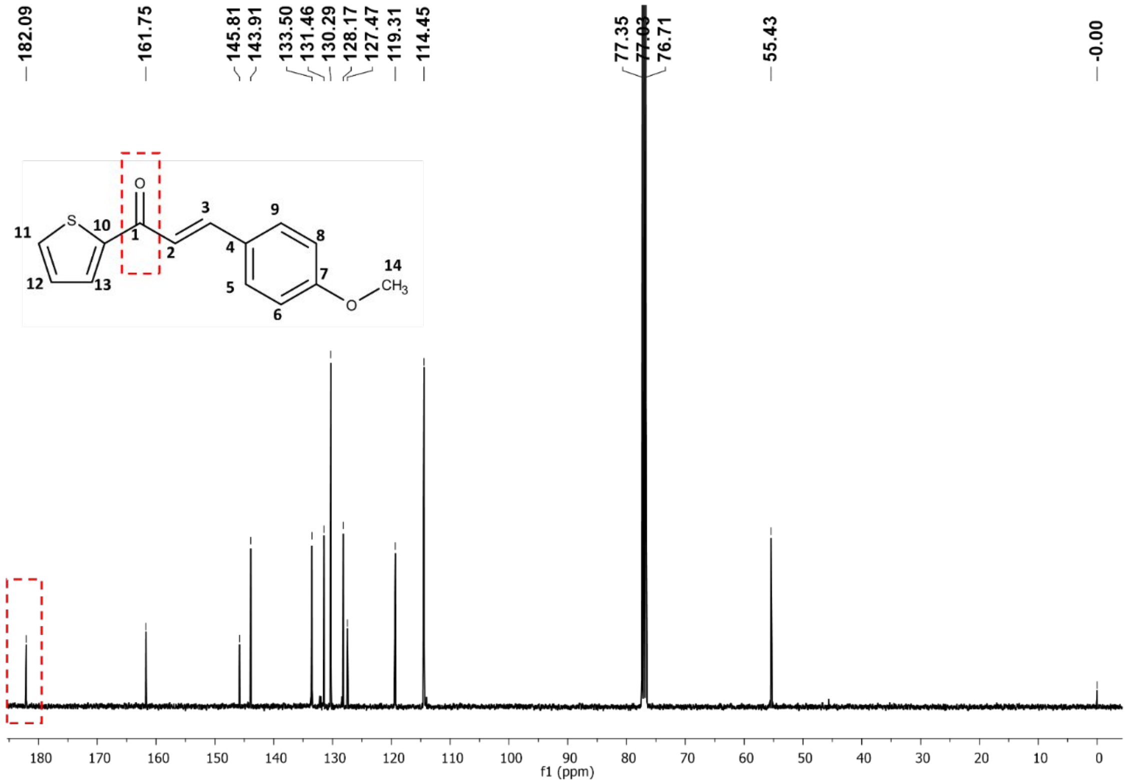

Figure S11. ^{1}H NMR spectrum ($CDCl_3$, 400 MHz) of Chal 6.

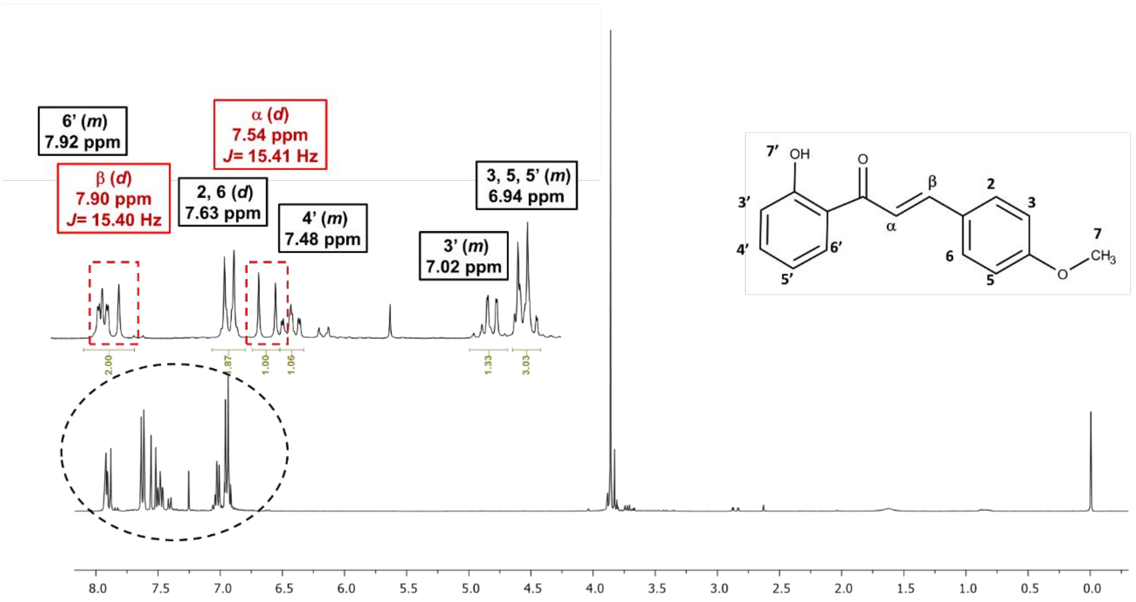

Figure S12. ^{13}C NMR spectrum ($CDCl_3$, 100 MHz) of Chal 6.

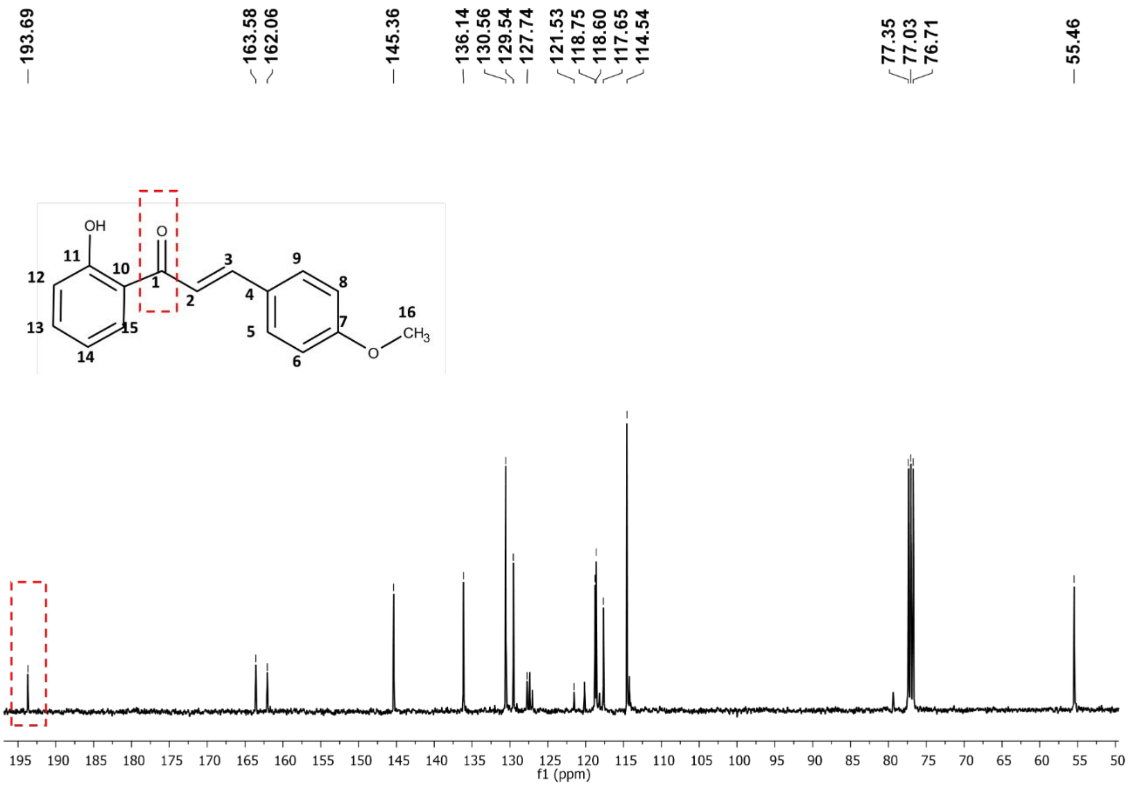

Figure S13. ^{1}H NMR spectrum ($CDCl_3$, 400 MHz) of Chal 7.

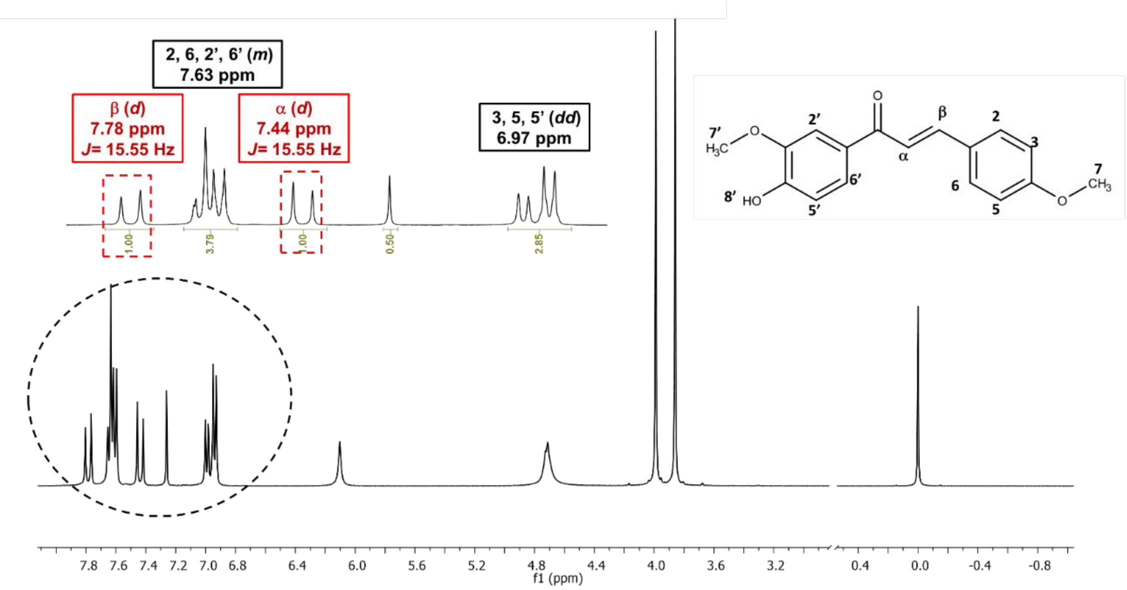

Figure S14. ^{13}C NMR spectrum ($CDCl_3$, 100 MHz) of Chal 7.

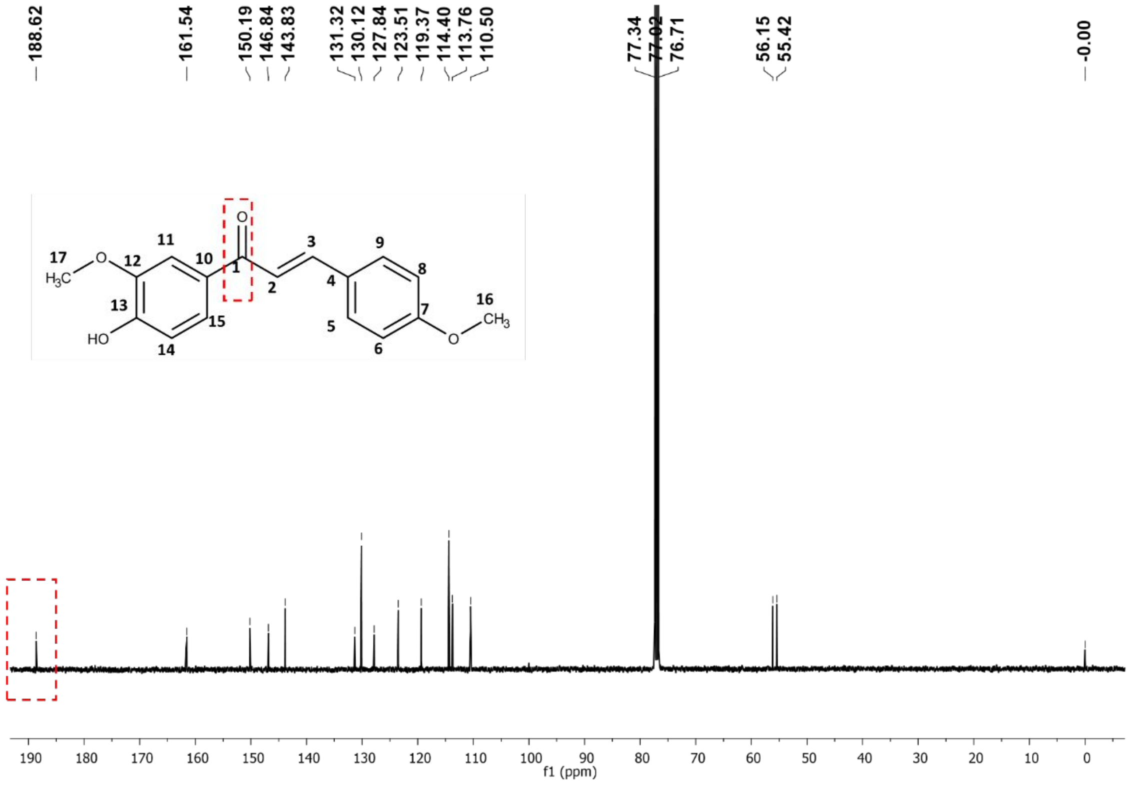

Figure S15. ^{1}H NMR spectrum ($CDCl_3$, 400 MHz) of Chal 8.

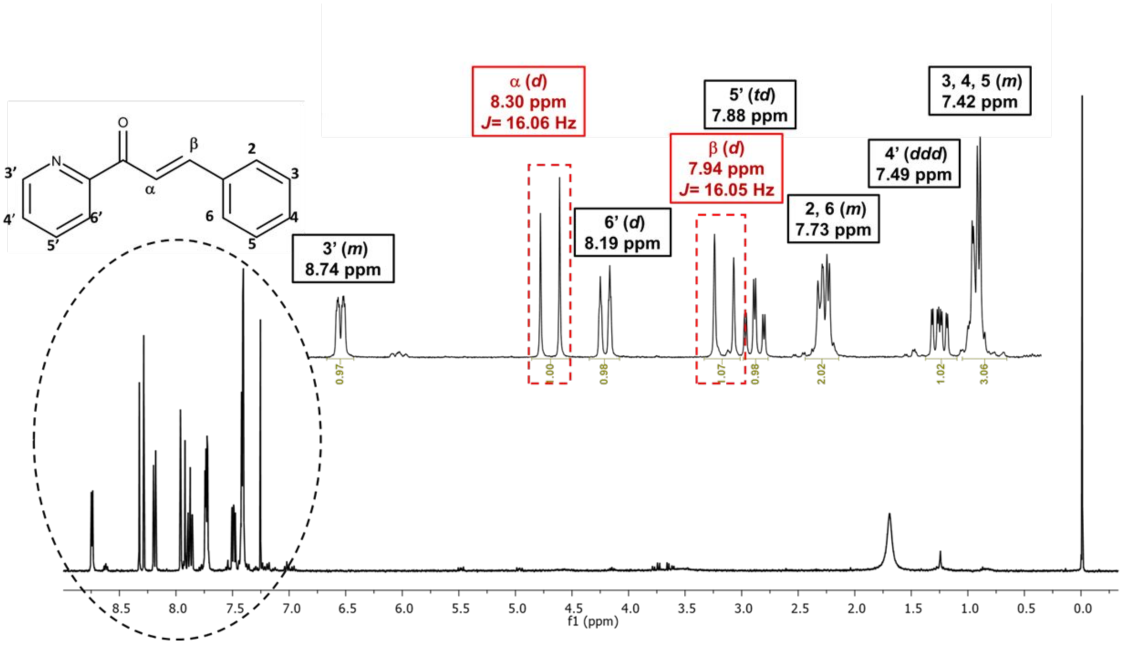

Figure S16. ^{13}C NMR spectrum ($CDCl_3$, 100 MHz) of Chal 8.

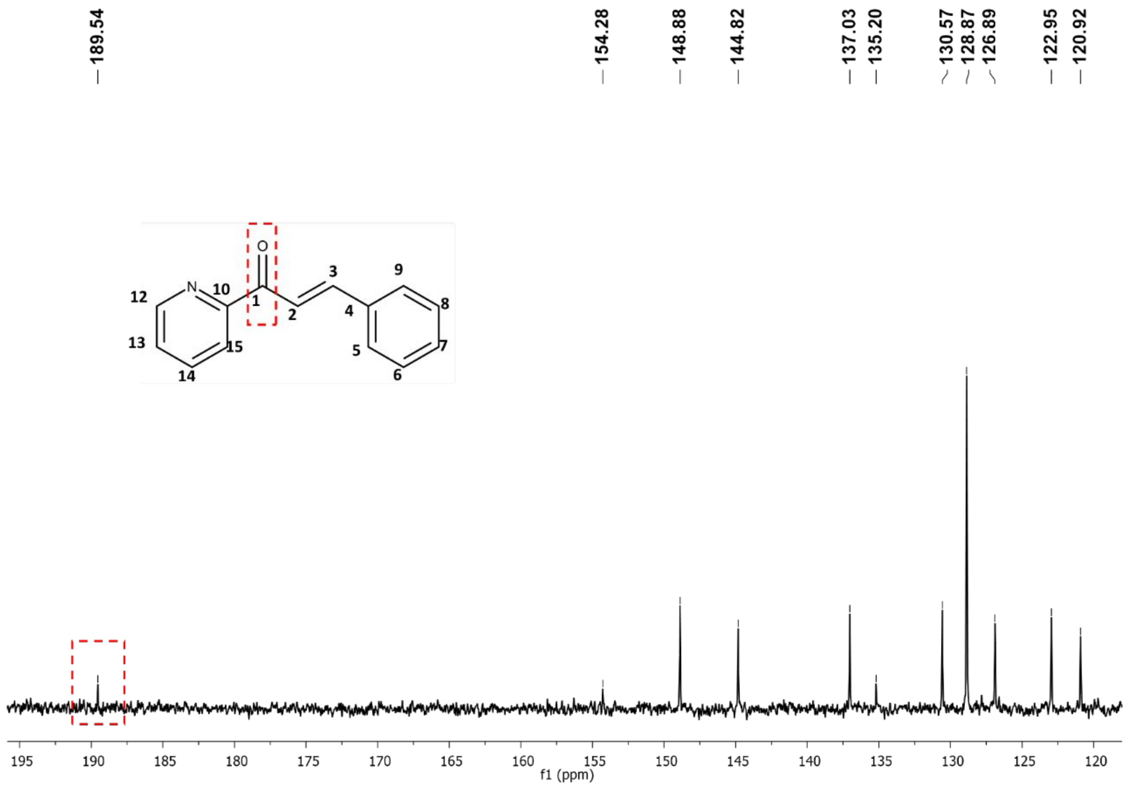

Figure S17. ^{1}H NMR spectrum ($CDCl_3$, 400 MHz) of Chal 9.

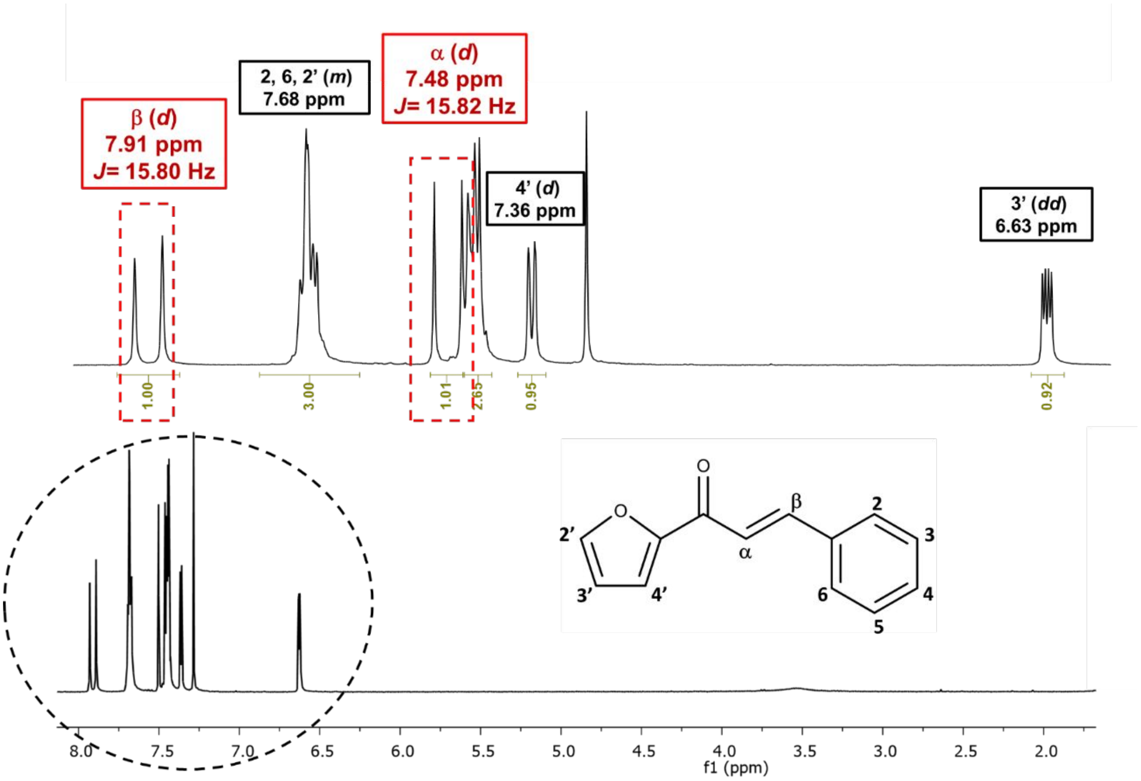

Figure S18. ^{13}C NMR spectrum ($CDCl_3$, 100 MHz) of Chal 9.

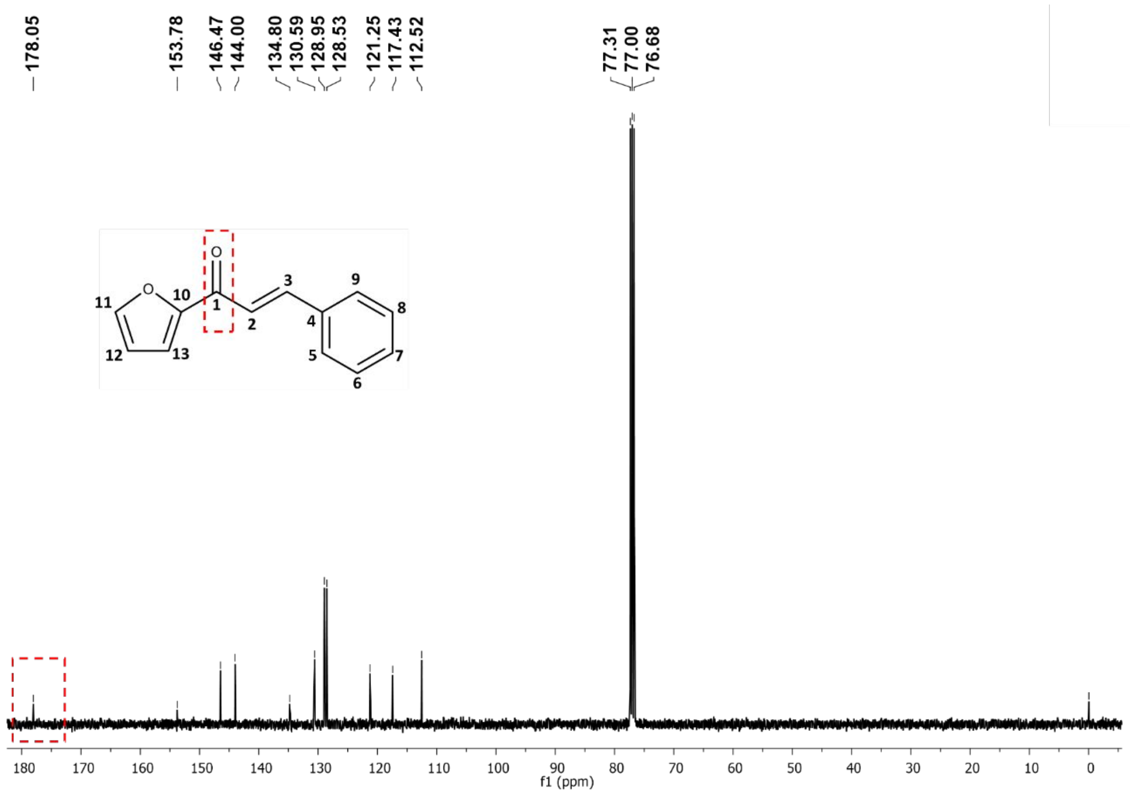

Figure S19. ^{1}H NMR spectrum ($CDCl_3$, 400 MHz) of Chal 10.

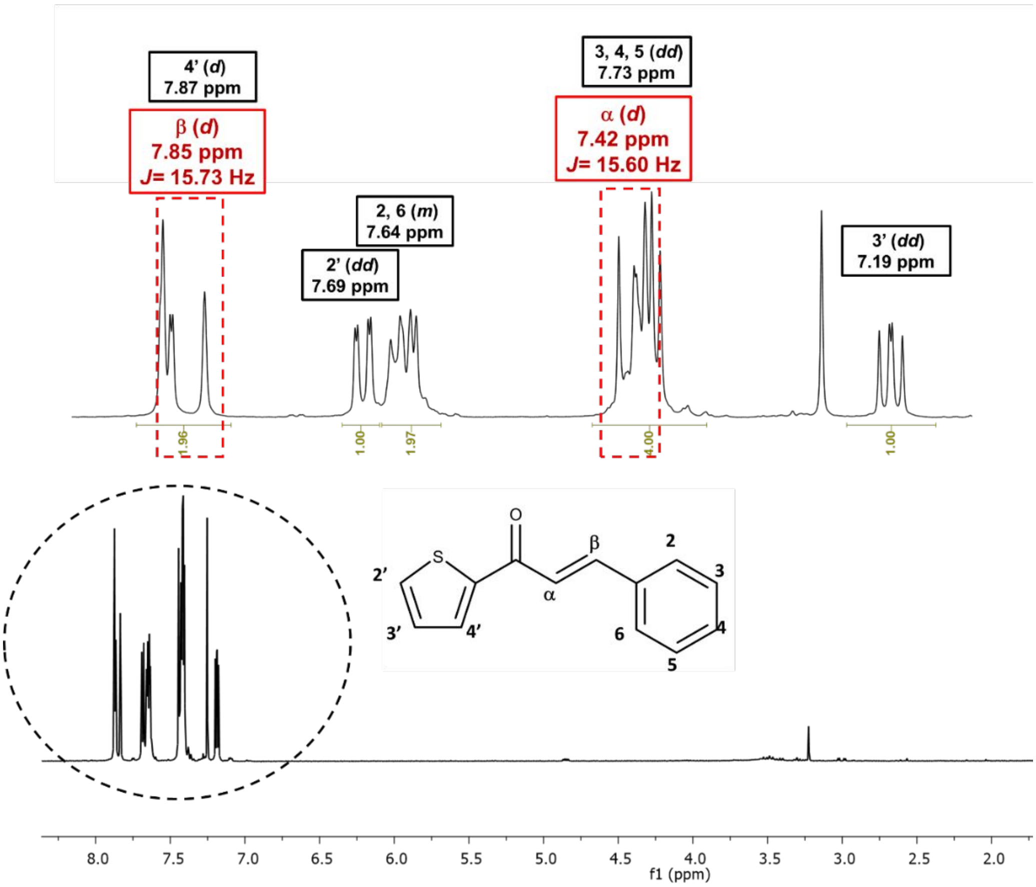

Figure S20. ^{13}C NMR spectrum ($CDCl_3$, 100 MHz) of Chal 10.

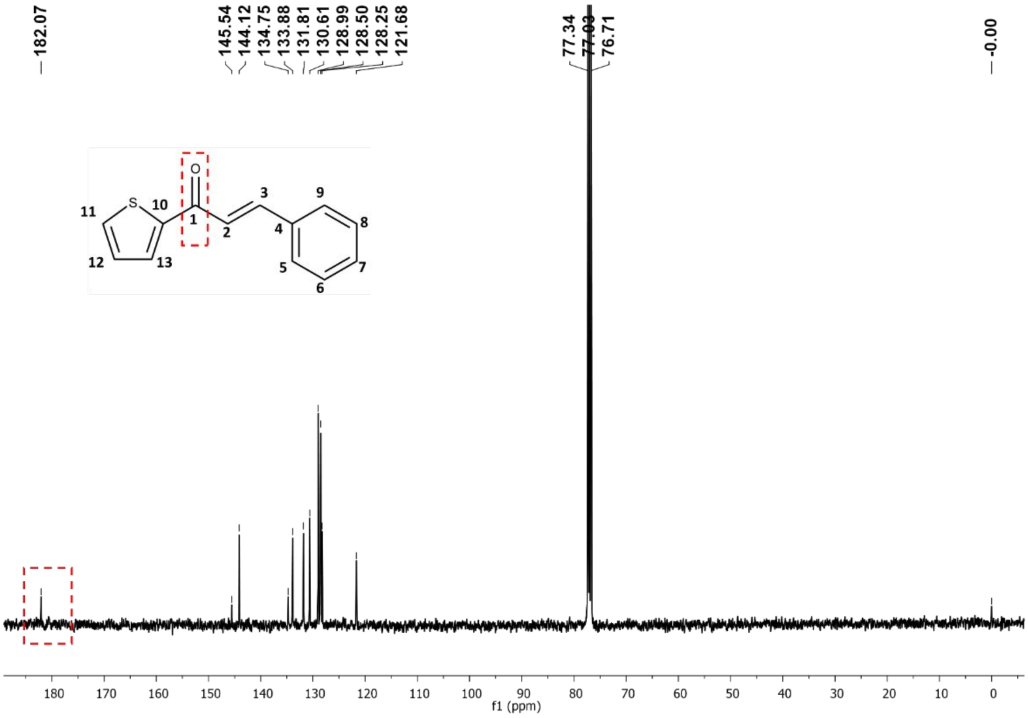

Figure S21. ^{1}H NMR spectrum ($CDCl_3$, 400 MHz) of Chal 11.

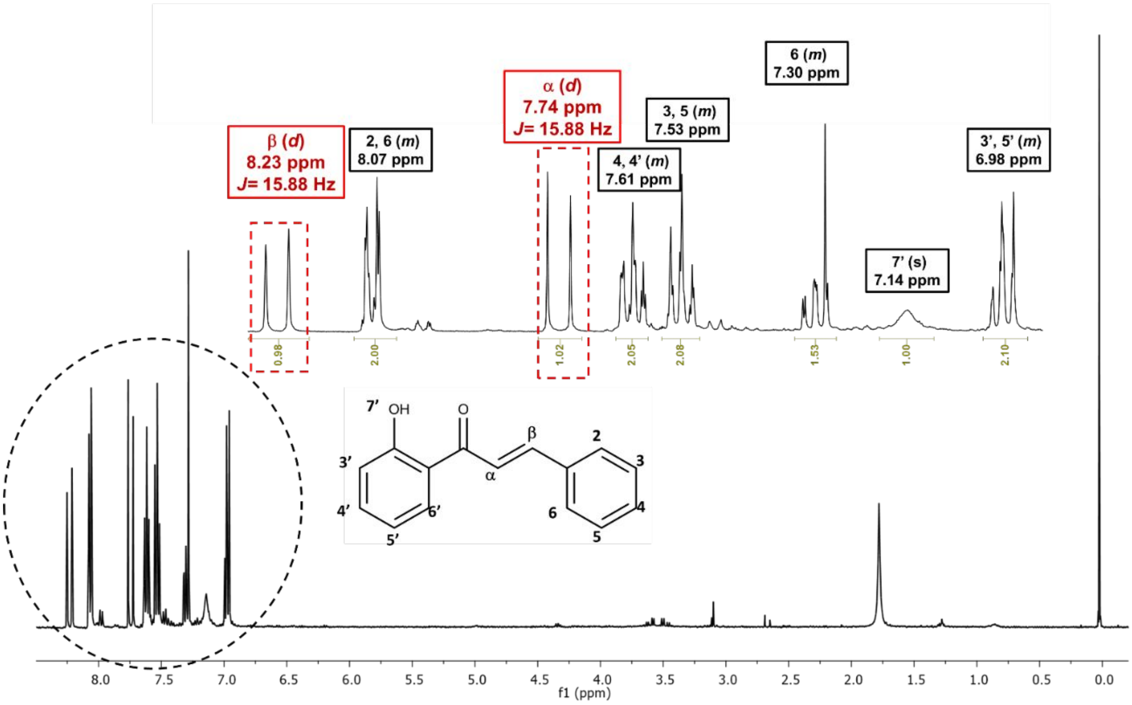

Figure S22. ^{13}C NMR spectrum ($CDCl_3$, 100 MHz) of Chal 11.

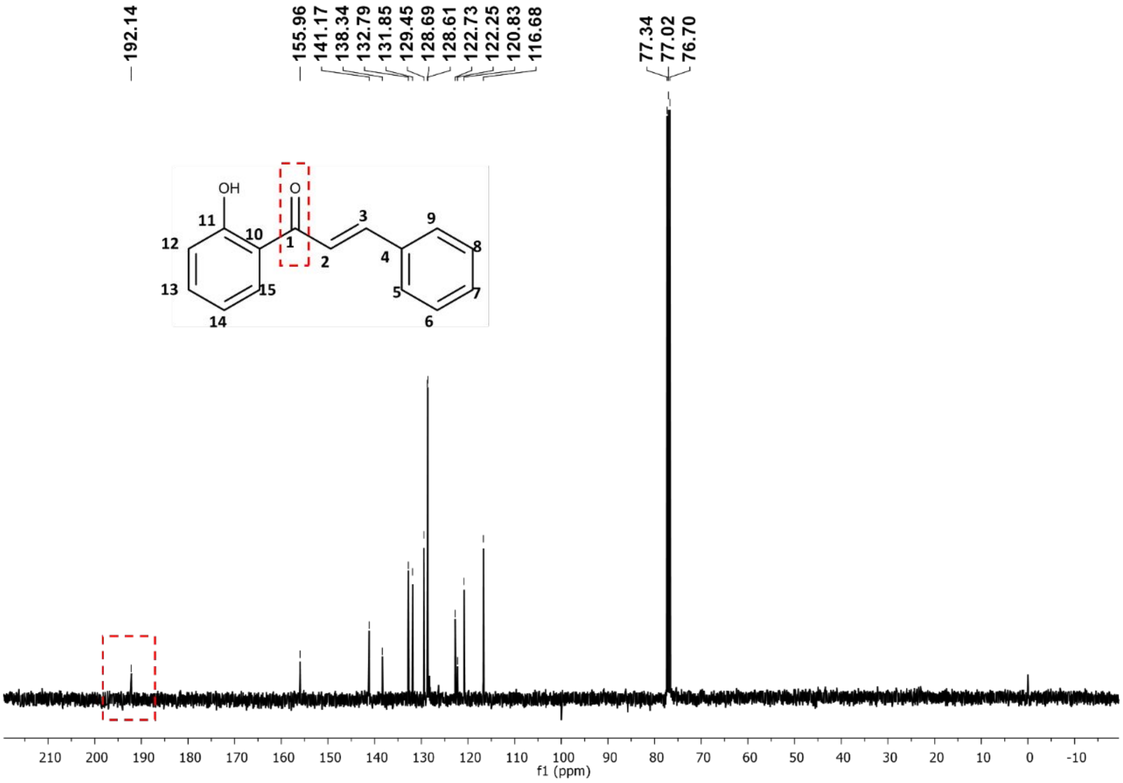

Figure S23. ^{1}H NMR spectrum ($CDCl_3$, 400 MHz) of Chal 12.

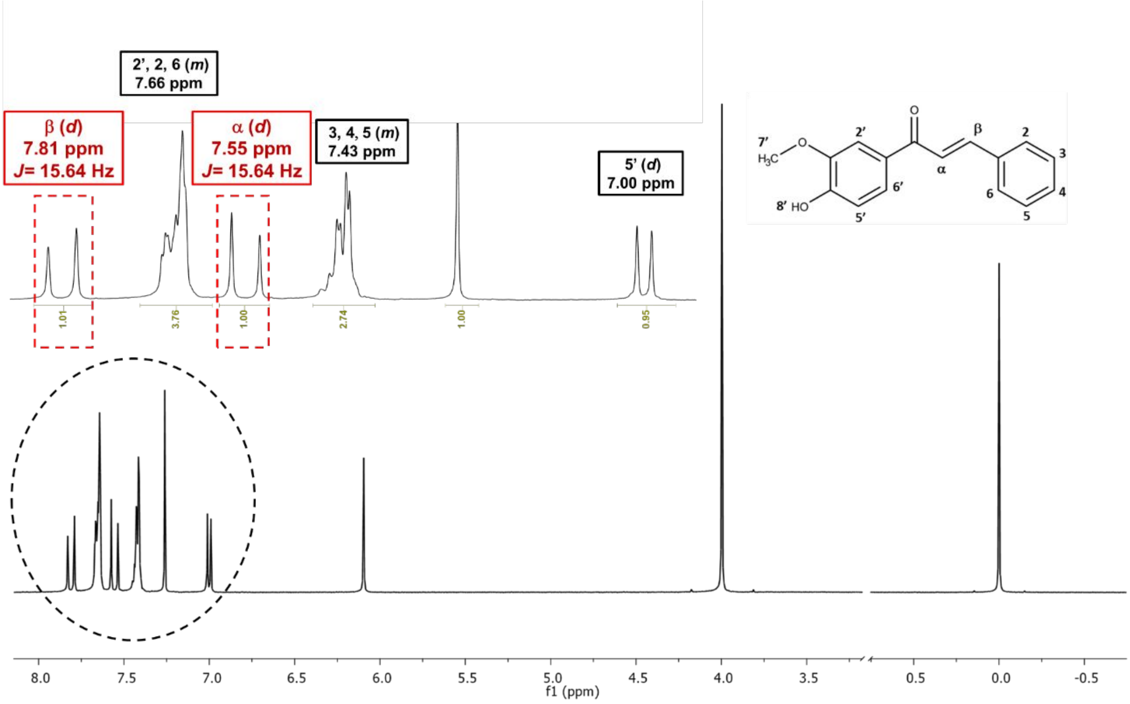

Figure S24. ^{13}C NMR spectrum ($CDCl_3$, 100 MHz) of Chal 12.

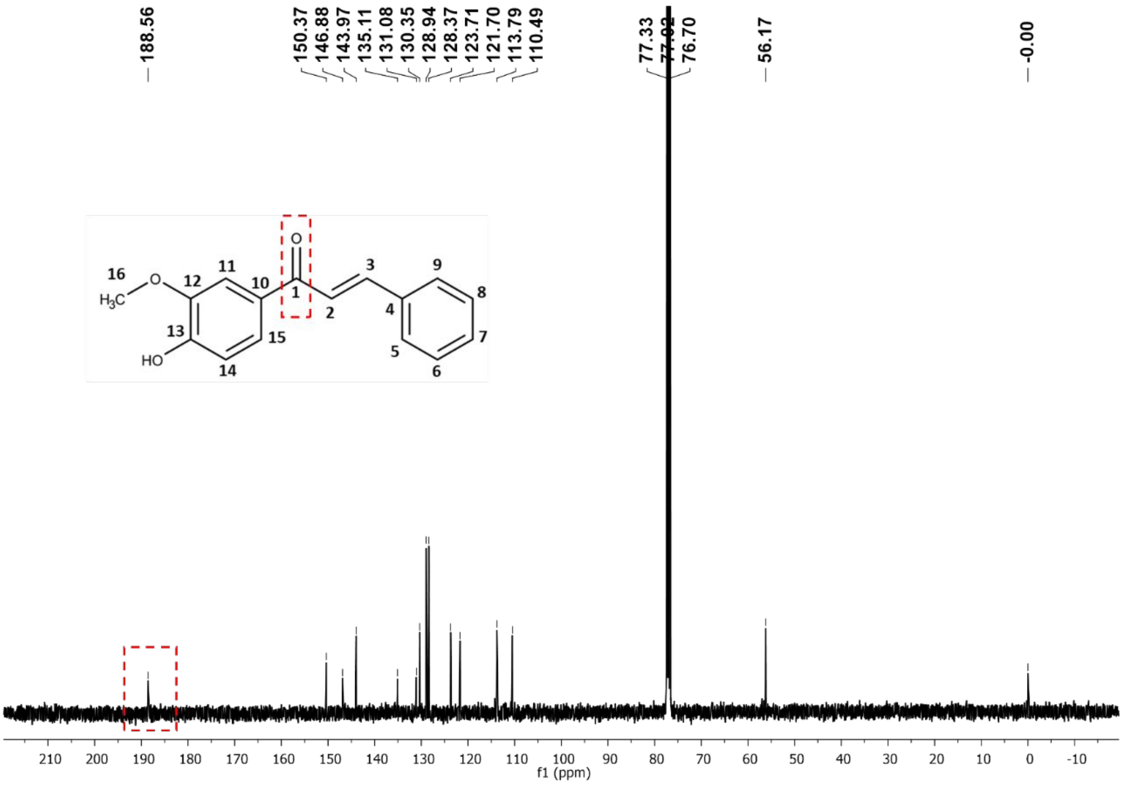

Figure S25. ^{1}H NMR spectrum ($CDCl_3$, 400 MHz) of Chal 13.

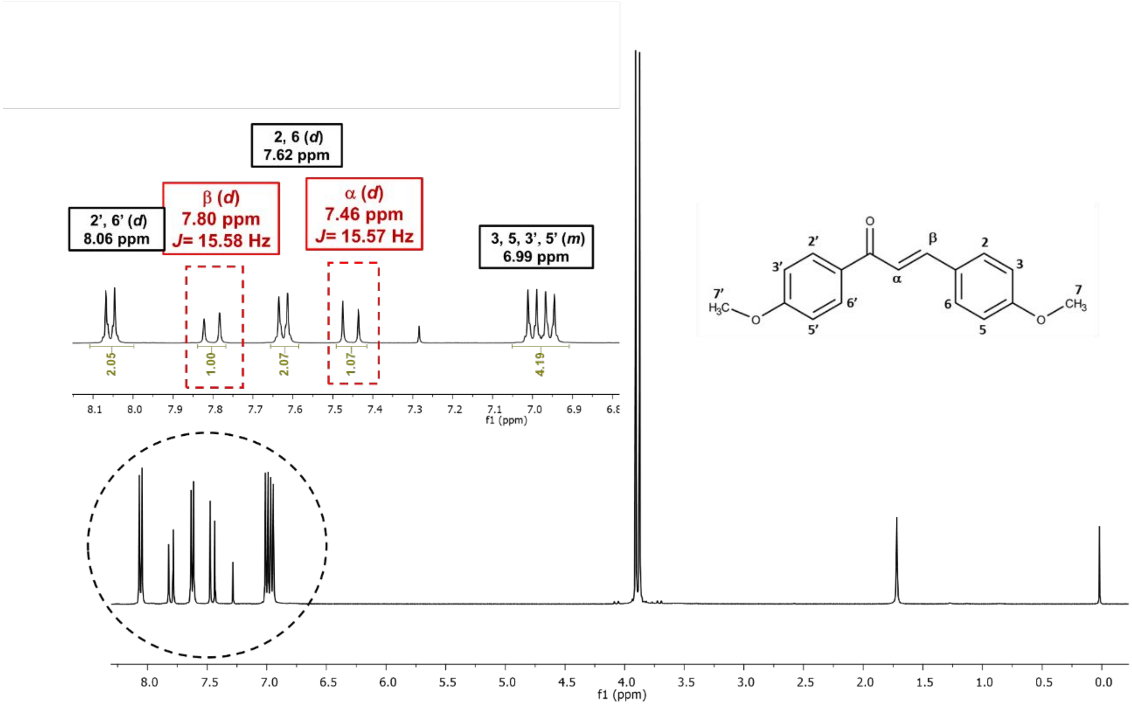

Figure S26. ^{13}C NMR spectrum ($CDCl_3$, 100 MHz) of Chal 13.

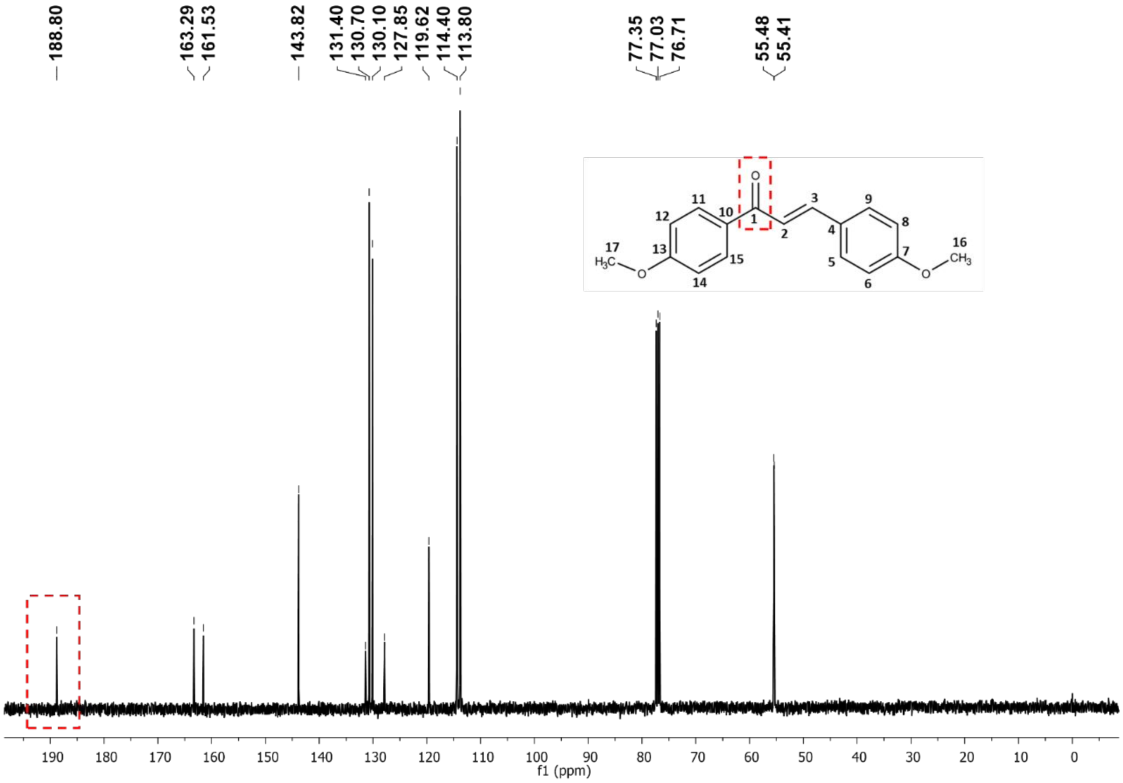

Figure S27. ^{1}H NMR spectrum ($CDCl_3$, 400 MHz) of Chal 14.

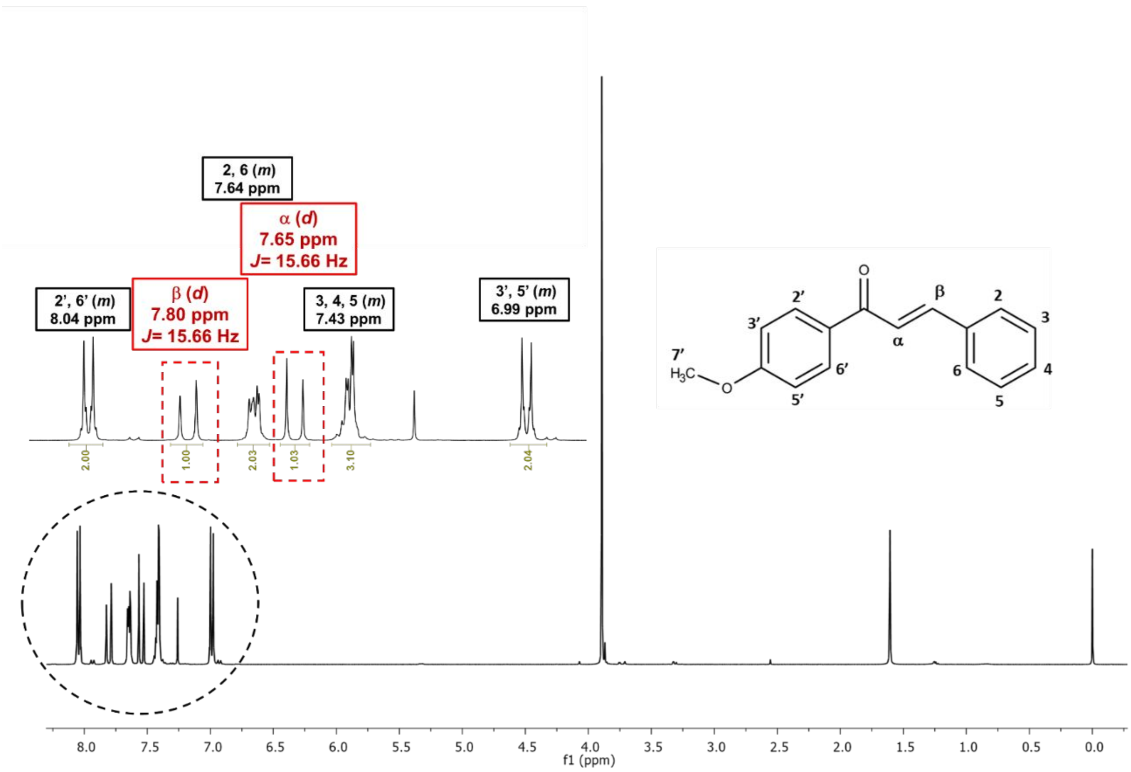

Figure S28. ^{13}C NMR spectrum ($CDCl_3$, 100 MHz) of Chal 14.

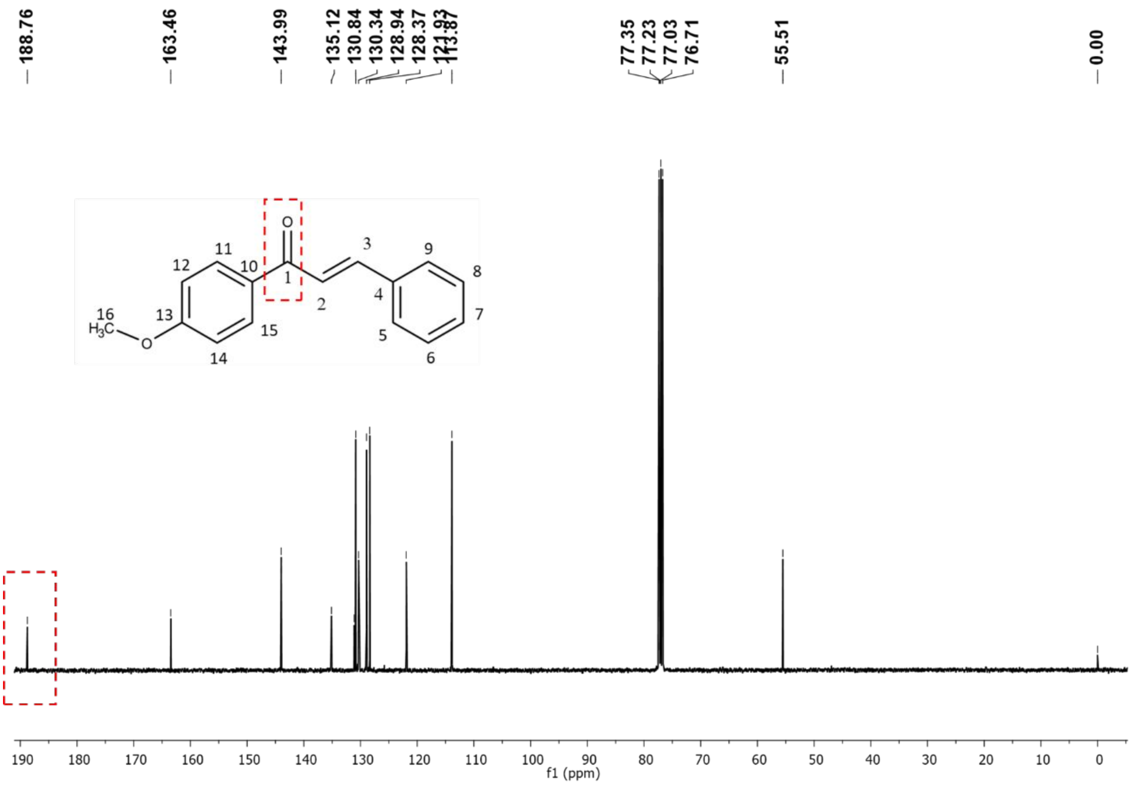

Figure S29. Cyclic voltammograms of compounds Chal 1 in BR buffer 0.04 M pH 2-10 and ethanol 1:1 (v/v), in the potential range of -0.2-1.4 V, with a scan rate of 0.1 V s-1

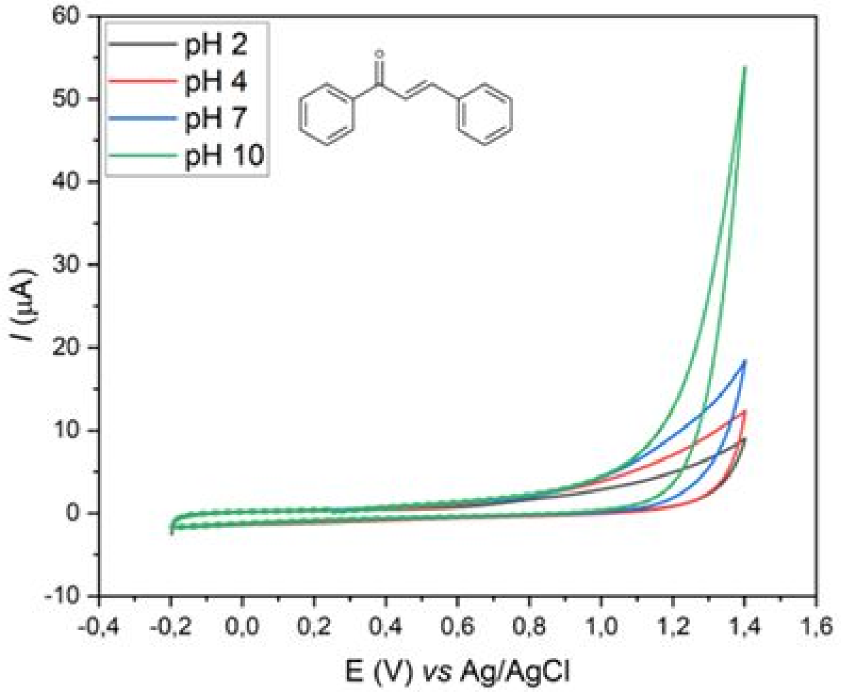

Figure S30. Relationships between pH vs E obtained from cyclic voltammograms.

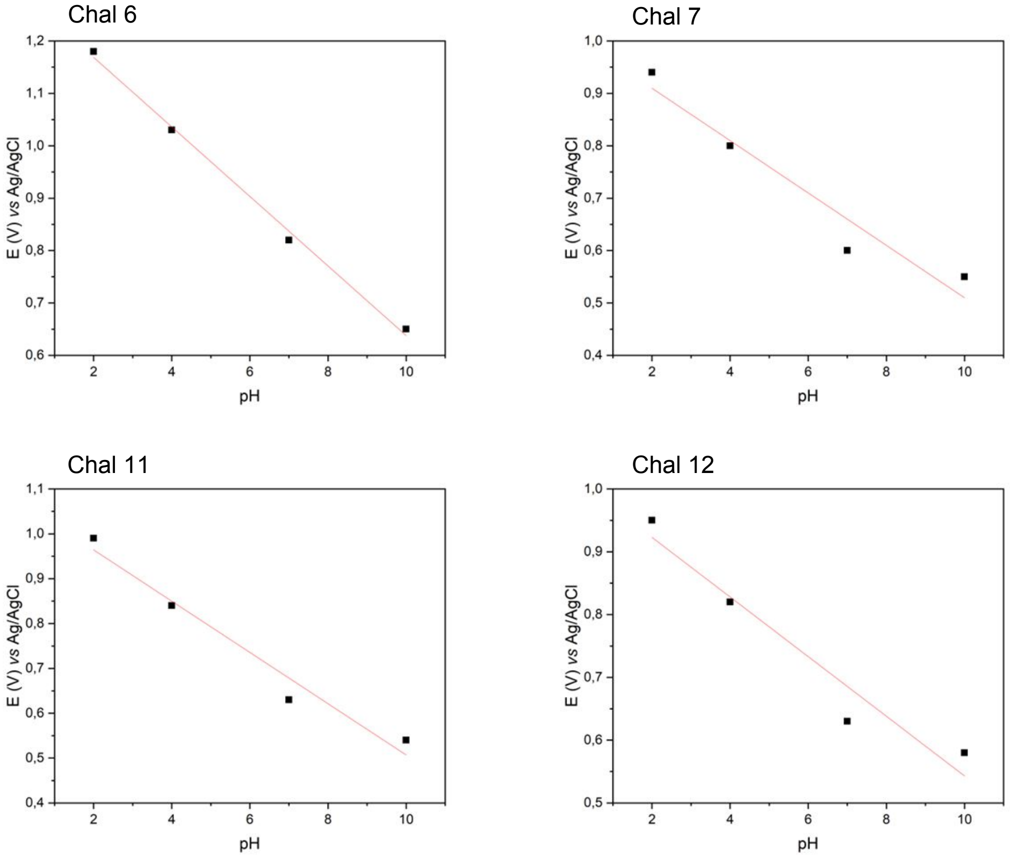

Figure S31. ^{1}H NMR of Chal 2 before and after exposure to UVA light.

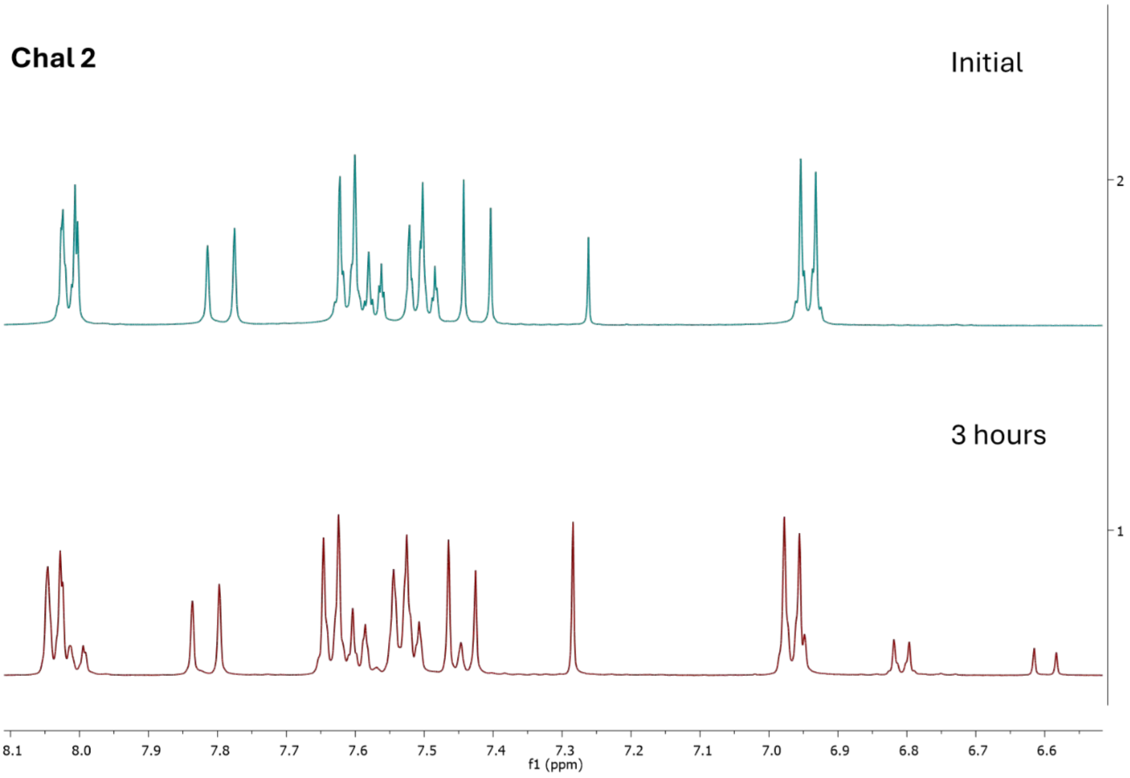

Figure S32. ^{1}H NMR of Chal 3 before and after exposure to UVA light.

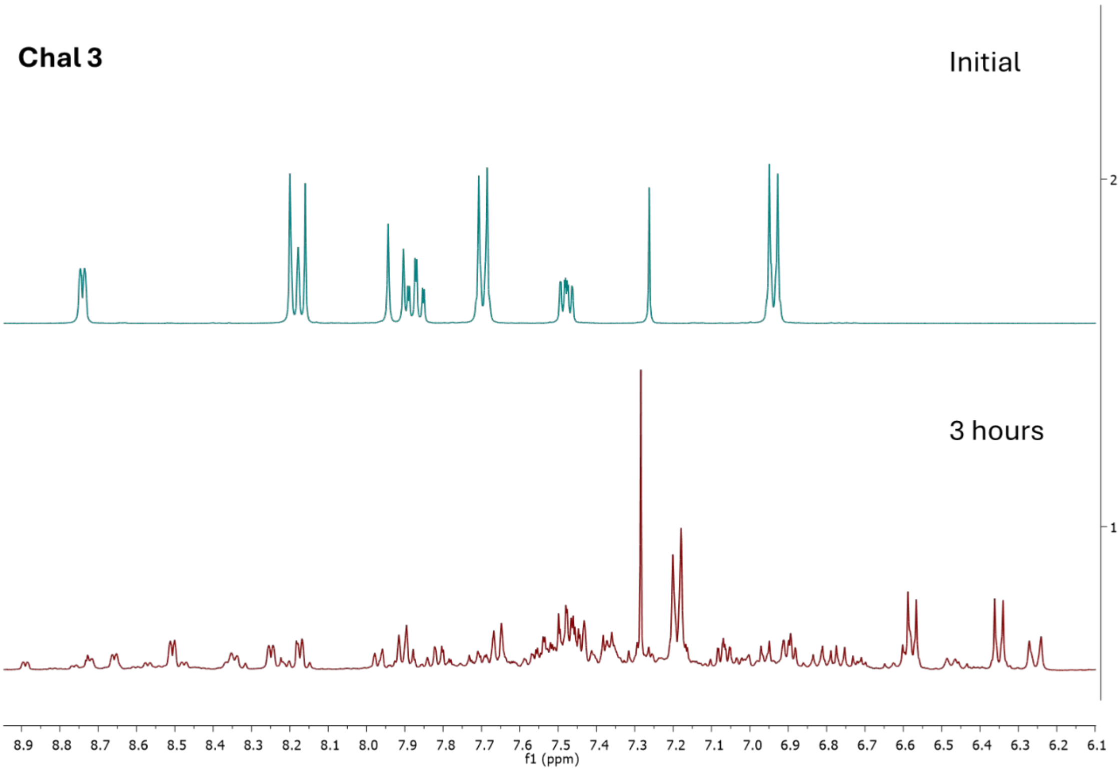

Figure S33. ^{1}H NMR of Chal 4 before and after exposure to UVA light.

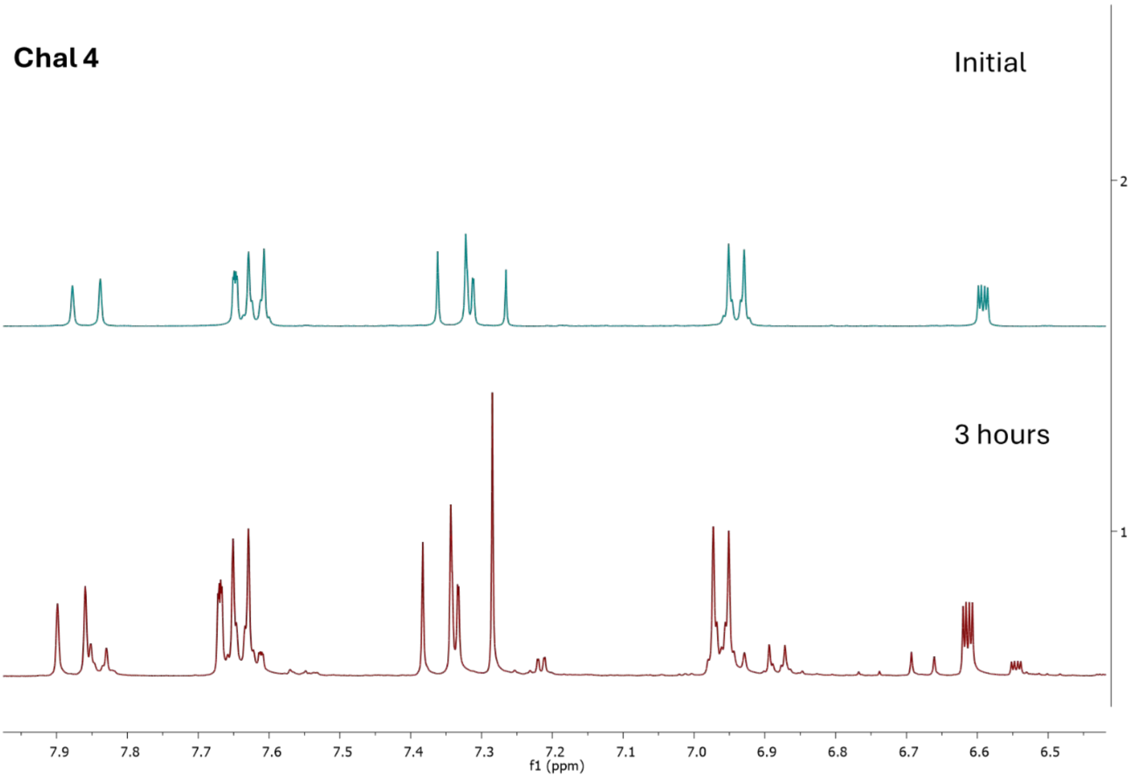

Figure S34. ^{1}H NMR of Chal 5 before and after exposure to UVA light.

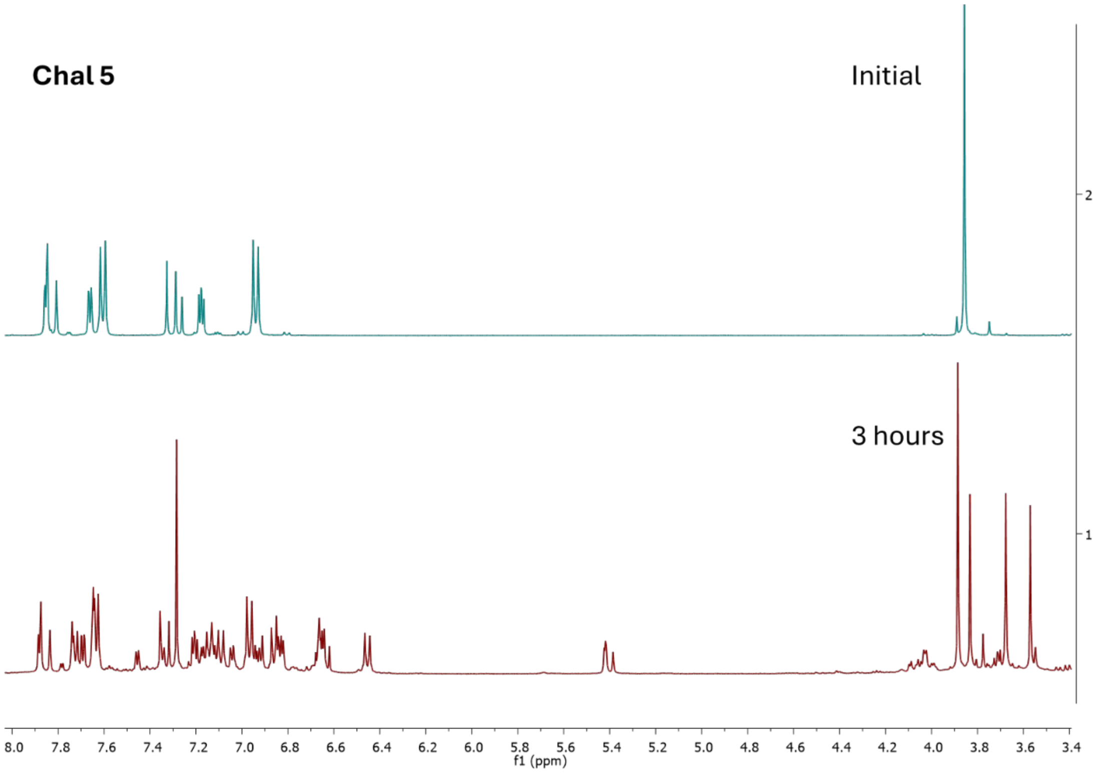

Figure S35. ^{1}H NMR of Chal 6 before and after exposure to UVA light.

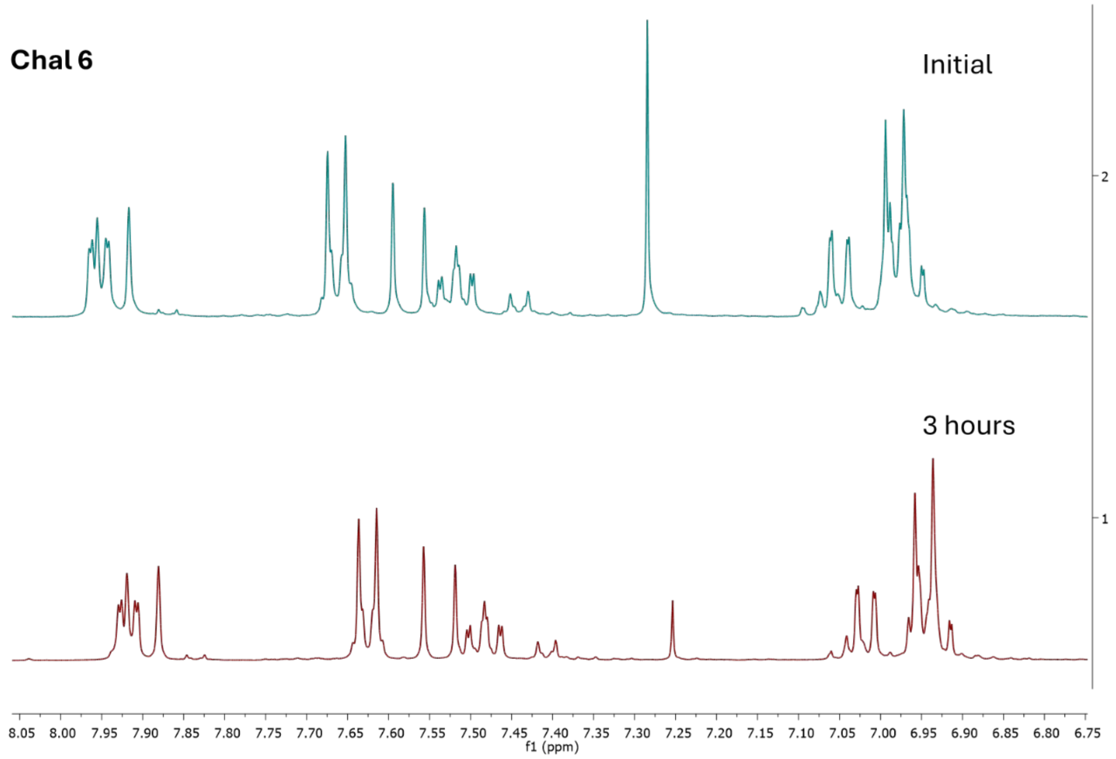

Figure S36. ¹H NMR of Chal 7 before and after exposure to UVA light.

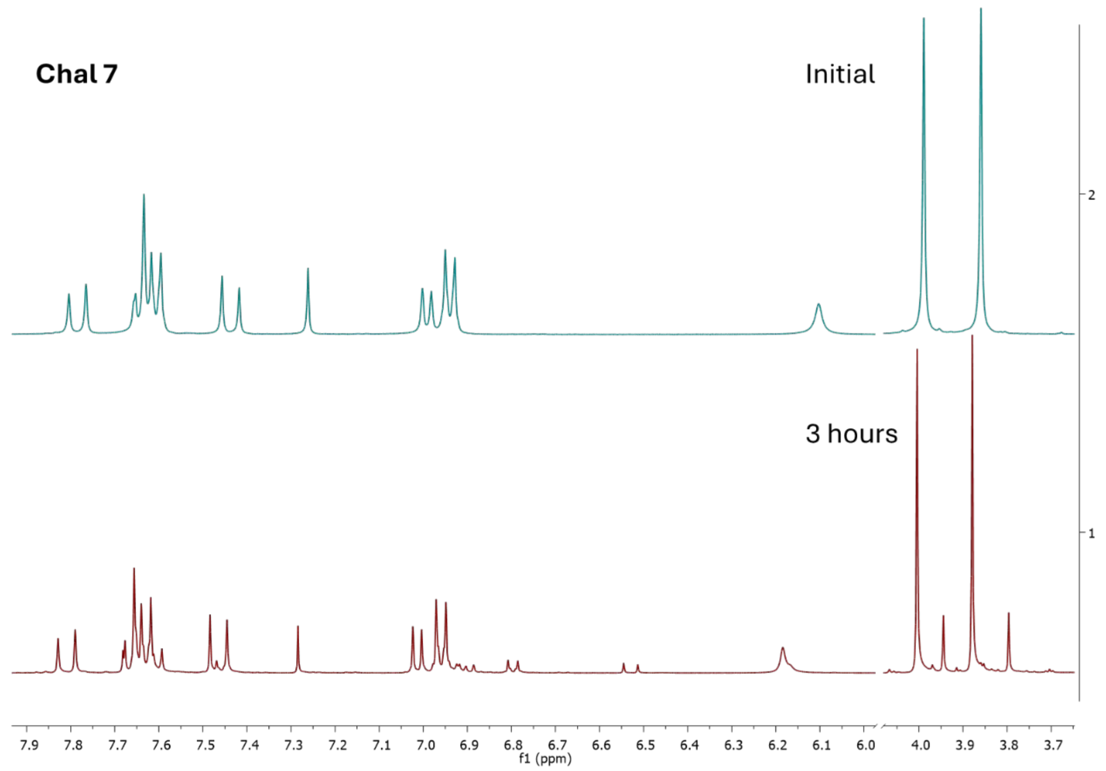

Figure S37. ¹H NMR of Chal 8 before and after exposure to UVA light.

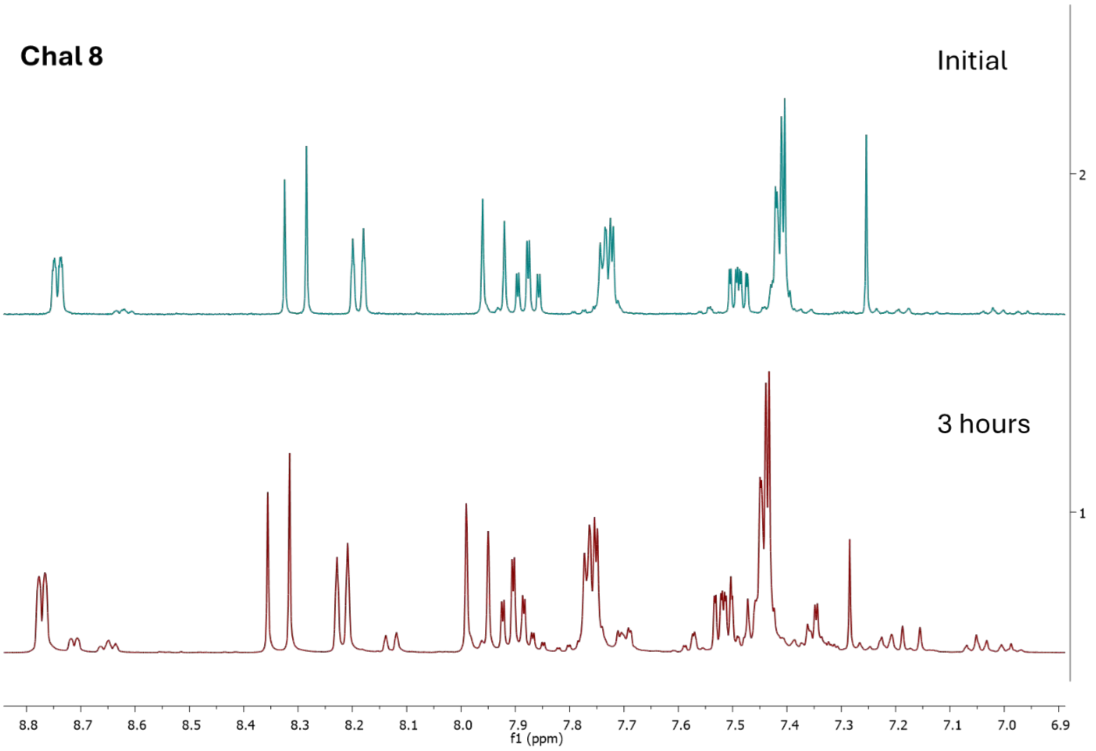

Figure S38. ^{1}H NMR of Chal 9 before and after exposure to UVA light.

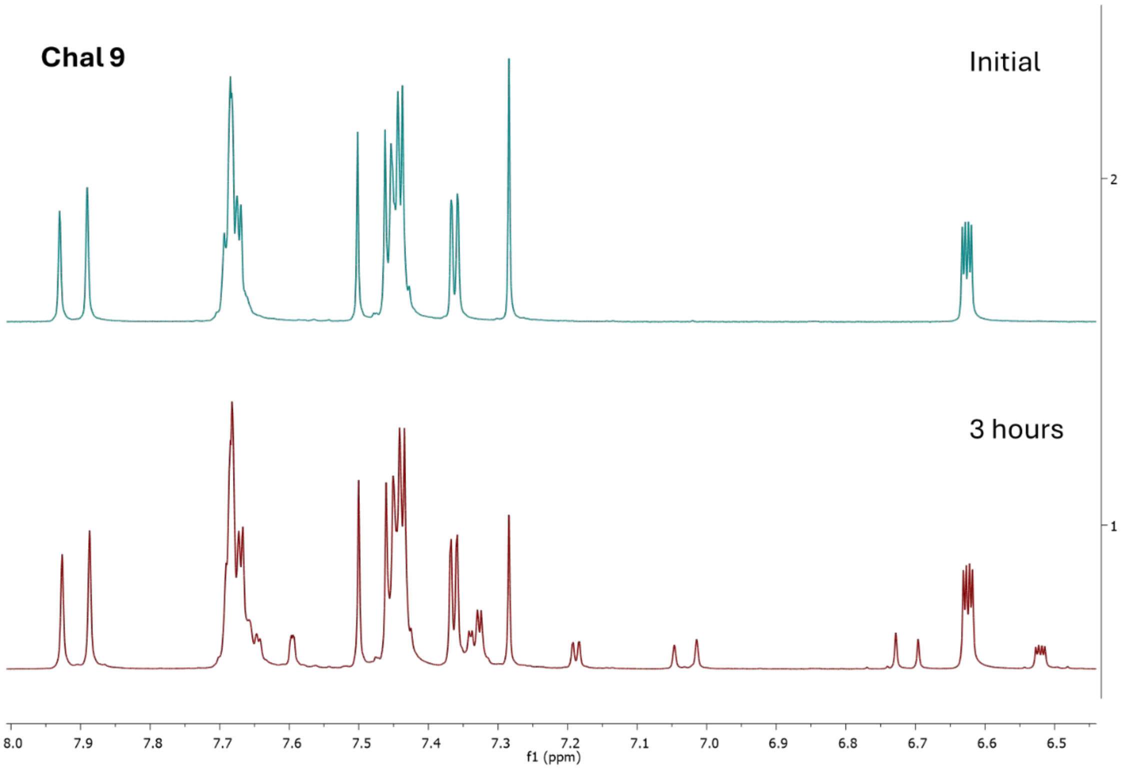

Figure S39. ^{1}H NMR of Chal 10 before and after exposure to UVA light.

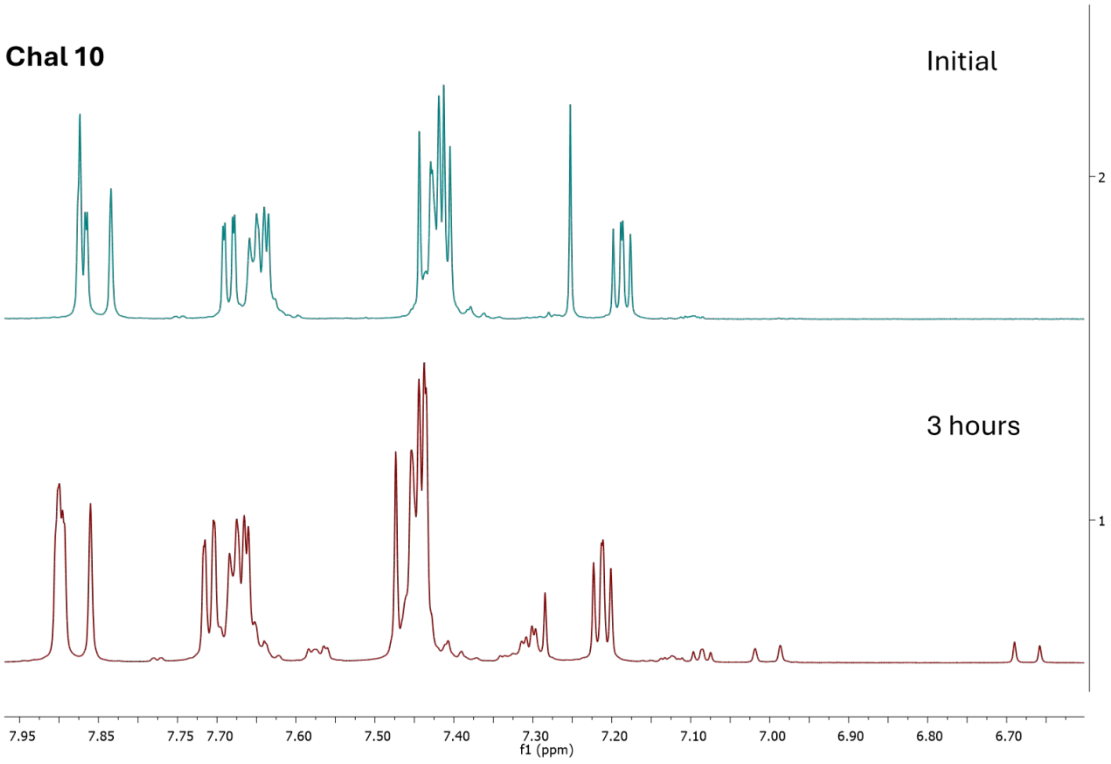

Figure S40. ^{1}H NMR of Chal 11 before and after exposure to UVA light.

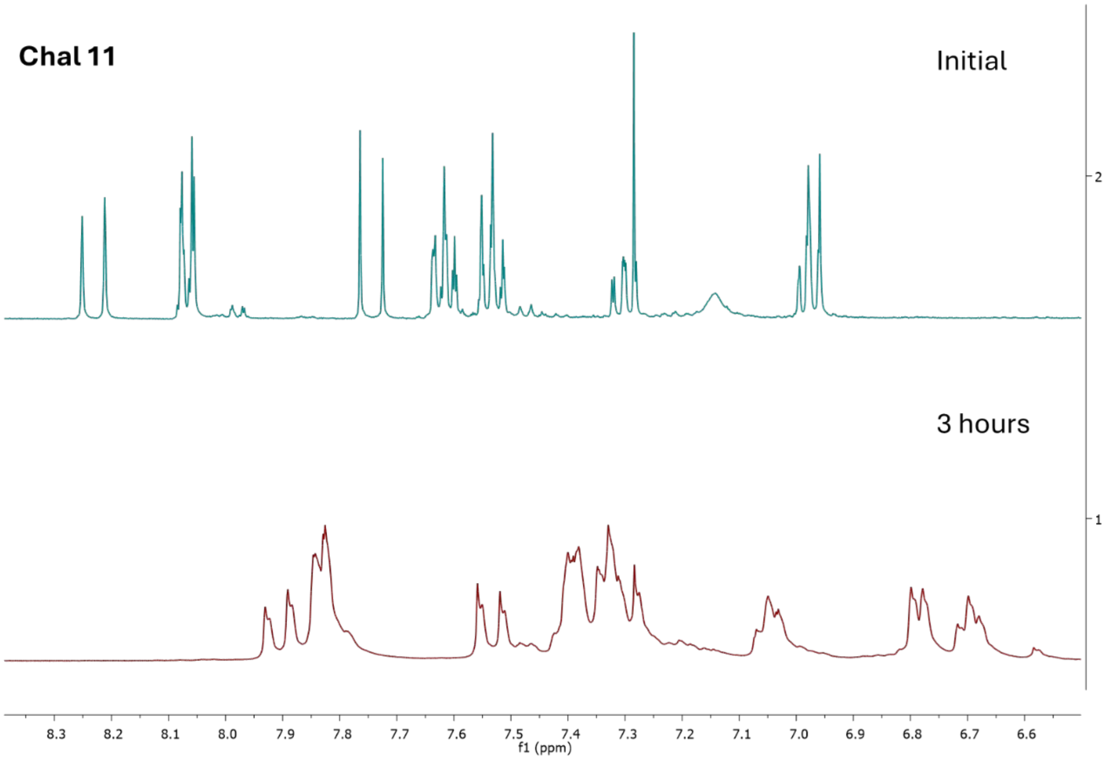

Figure S41. ^{1}H NMR of Chal 12 before and after exposure to UVA light.

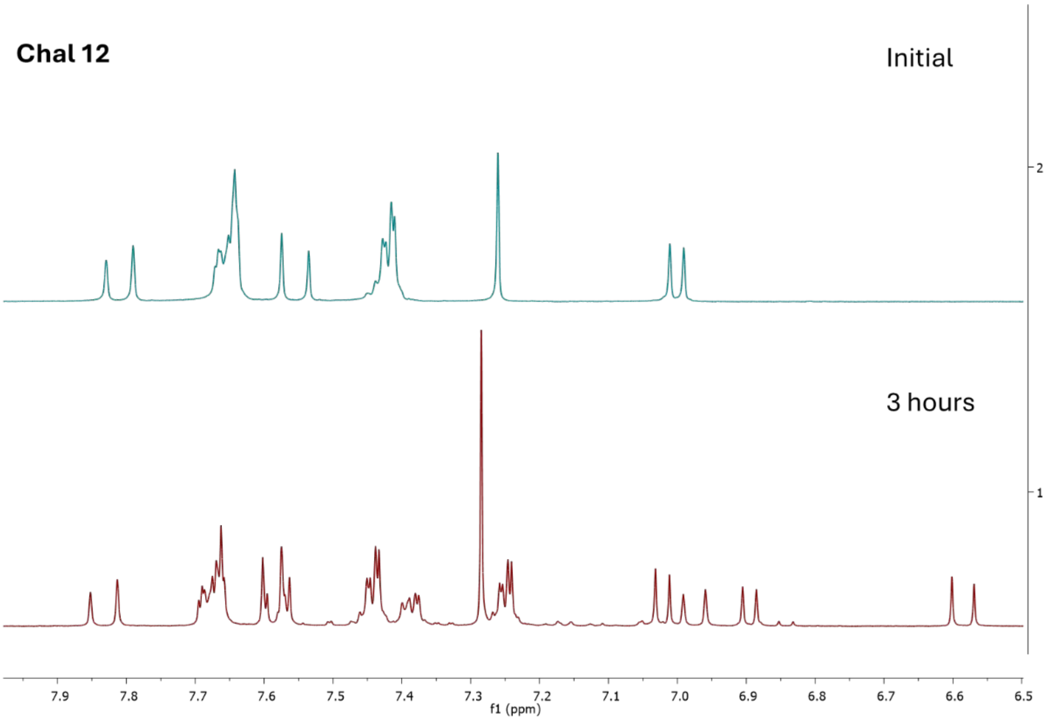

Figure S42. ^{1}H NMR of Chal 13 before and after exposure to UVA light.

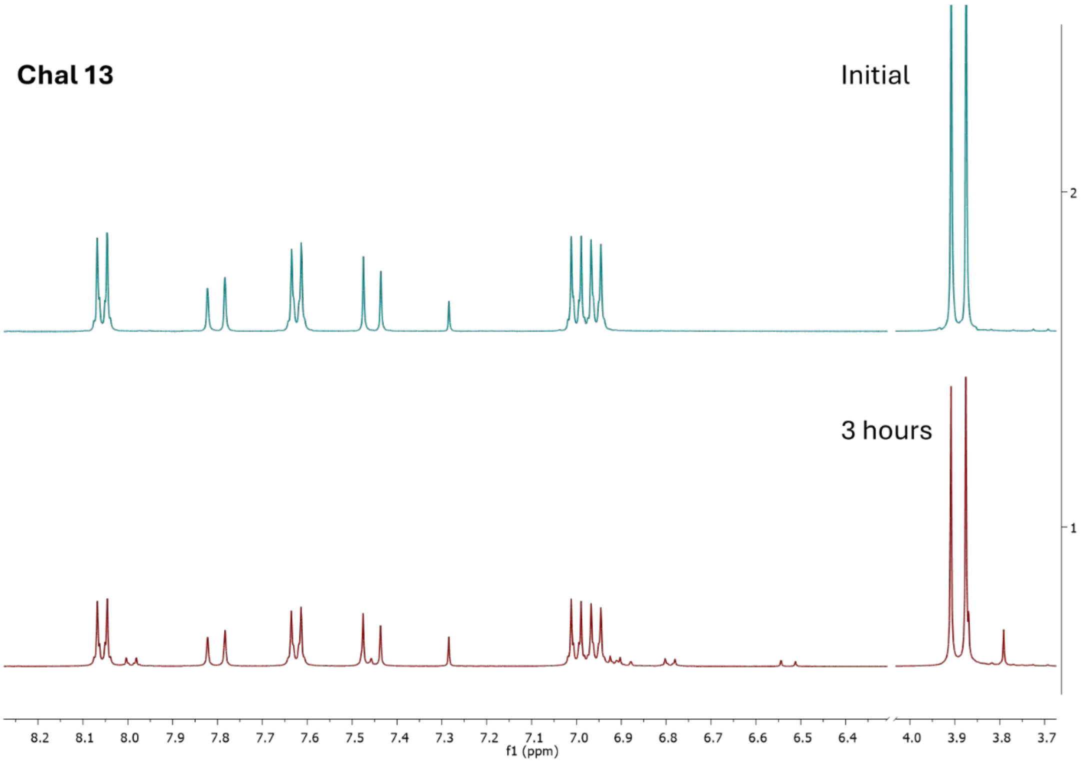

Figure S43. ^{1}H NMR of Chal 14 before and after exposure to UVA light.

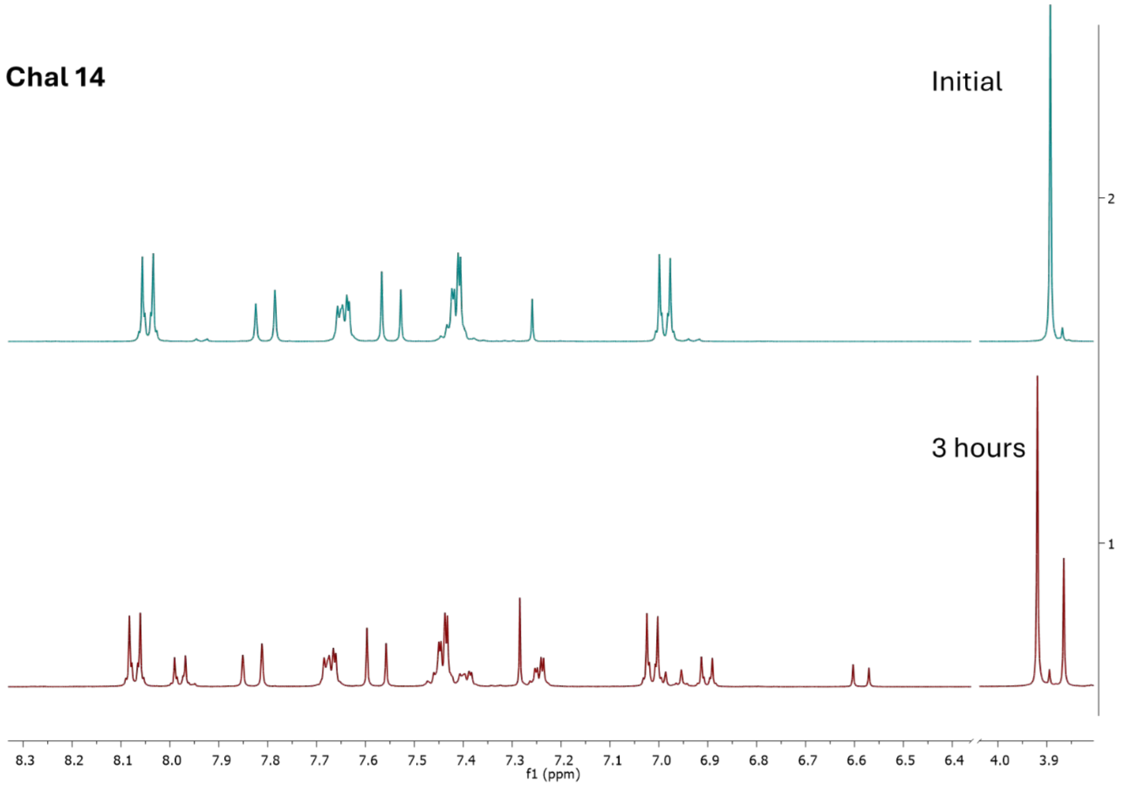

Figure S44. Kinetics of degradation of Chal 7 monitored by ¹H NMR under UV irradiation.

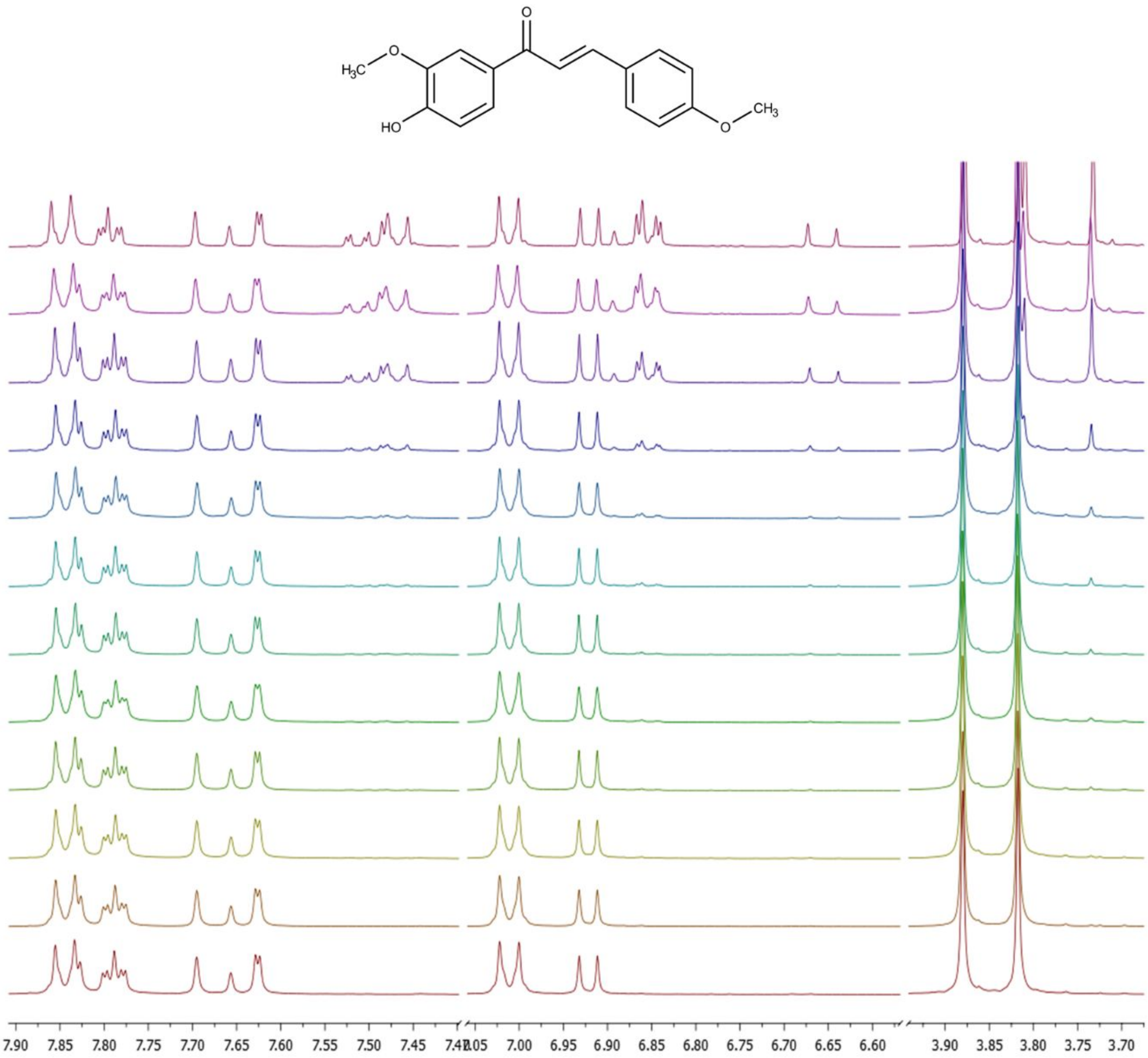

Figure S45. Kinetics of degradation of Chal 9 monitored by ^{1}H NMR under UV irradiation.

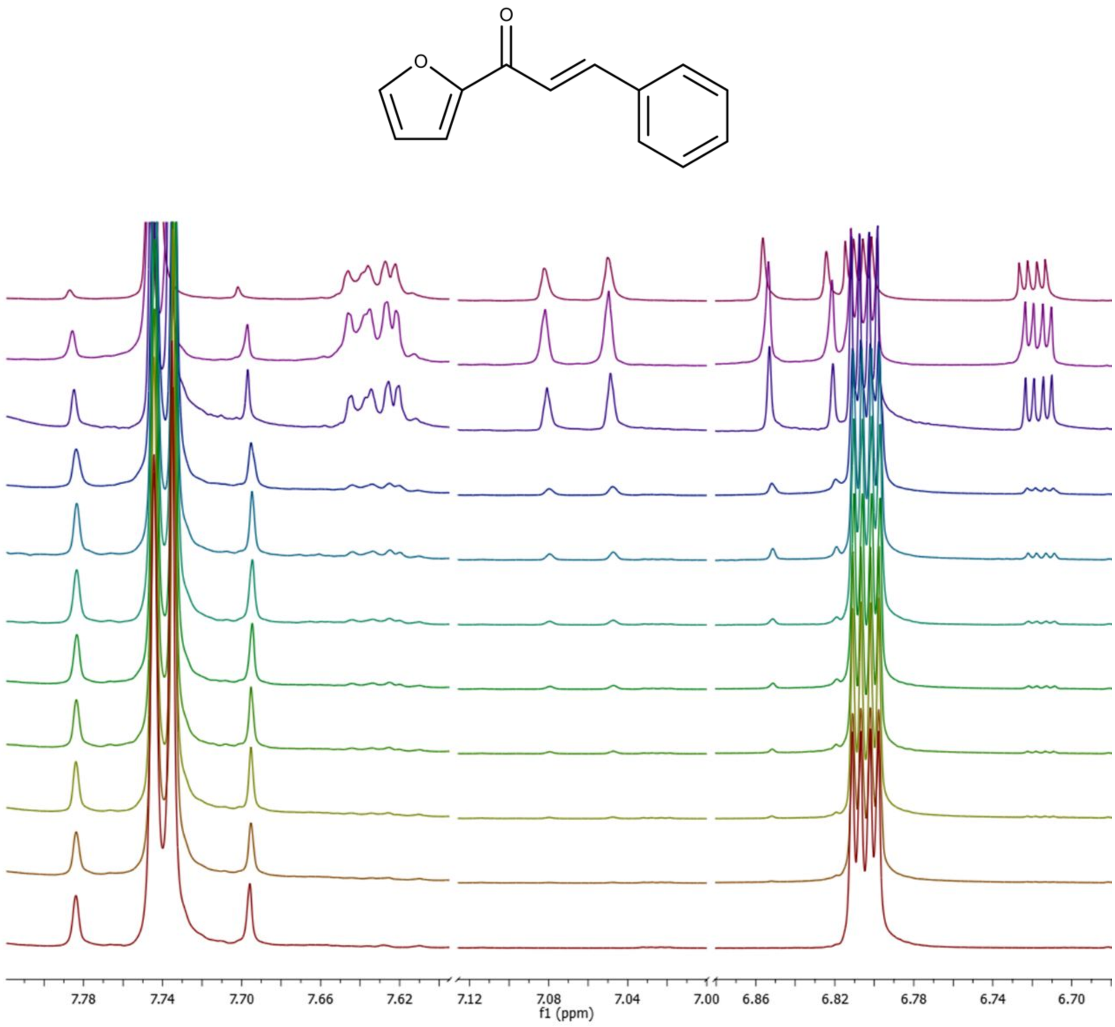

Figure S46. Kinetics of degradation of Chal 10 monitored by ^{1}H NMR under UV irradiation.

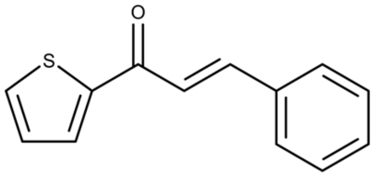

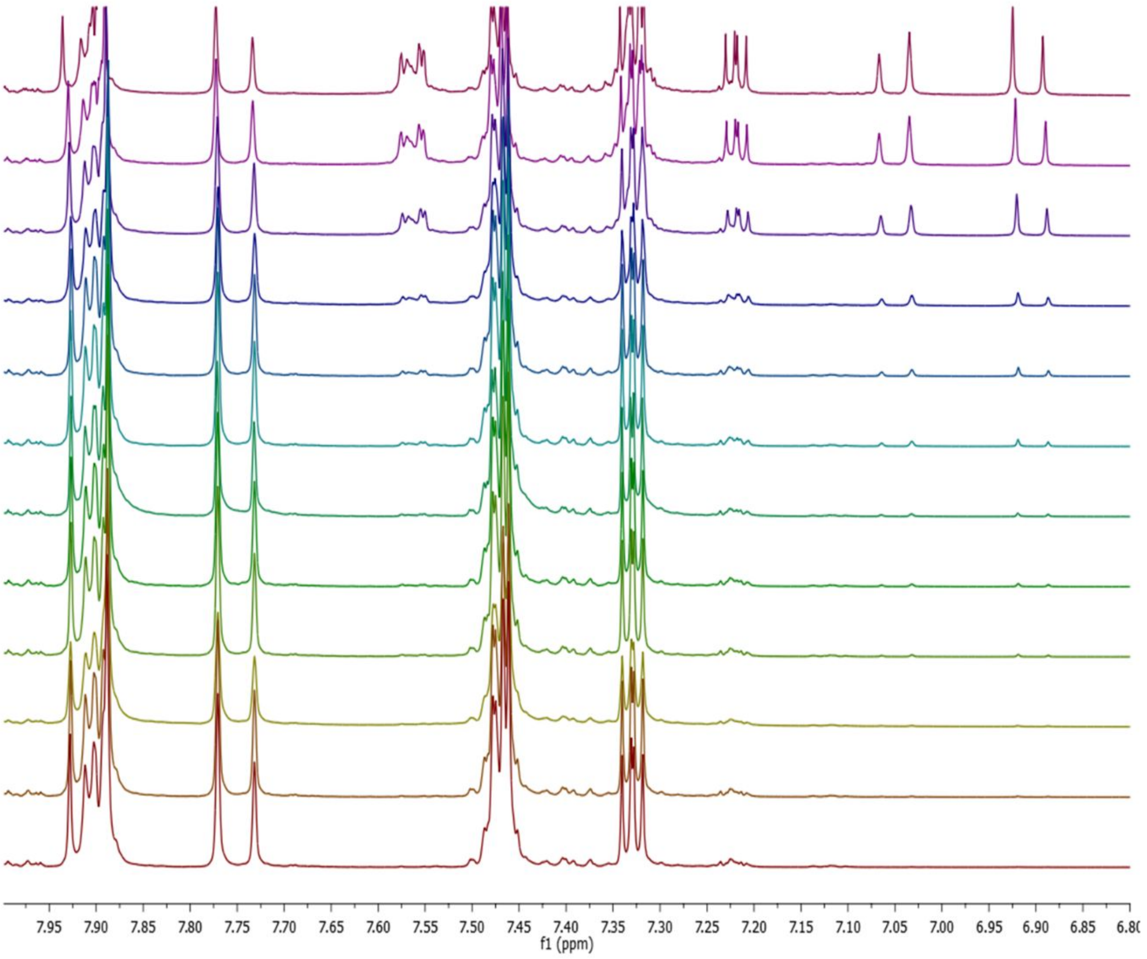

Supplement: Supplementary file 1 [file ao5c08934_si_001.pdf]
